# Estimating biological accuracy of DSM for attention deficit/hyperactivity disorder based on multivariate analysis for small samples

**DOI:** 10.7717/peerj.7074

**Published:** 2019-06-12

**Authors:** Dimitri M. Abramov, Vladimir V. Lazarev, Saint Clair Gomes Junior, Carlos Alberto Mourao-Junior, Monique Castro-Pontes, Carla Q. Cunha, Leonardo C. deAzevedo, Evelyne Vigneau

**Affiliations:** 1 Laboratory of Neurobiology and Clinical Neurophysiology, National Institute of Women, Children and Adolescents’ Health Fernandes Figueira, Oswaldo Cruz Foundation, Rio de Janeiro, Brazil; 2 Clinical Research Unit, National Institute of Women, Children, and Adolescents’ Health Fernandes Figueira, Oswaldo Cruz Foundation, Rio De Janeiro, Brazil; 3 Laboratoy of Psychophysiology, Institute of Biological Sciences, Federal University of Juiz de Fora, Juiz de Fora, Brazil; 4 StatSC, Oniris, INRA, Nantes, France

**Keywords:** ADHD, Diagnostic, Diagnostic and statistical manual of mental disorders, Multivariate analysis, Clustering of variables around latent components, Event-related potentials, Attentional networks test

## Abstract

**Objective:**

To estimate whether the “Diagnostic and Statistical Manual of Mental Disorders” (DSM) is biologically accurate for the diagnosis of Attention Deficit/ Hyperactivity Disorder (ADHD) using a biological-based classifier built by a special method of multivariate analysis of a large dataset of a small sample (much more variables than subjects), holding neurophysiological, behavioral, and psychological variables.

**Methods:**

Twenty typically developing boys and 19 boys diagnosed with ADHD, aged 10–13 years, were examined using the Attentional Network Test (ANT) with recordings of event-related potentials (ERPs). From 774 variables, a reduced number of latent variables (LVs) were extracted with a clustering of variables method (CLV), for further reclassification of subjects using the k-means method. This approach allowed a multivariate analysis to be applied to a significantly larger number of variables than the number of cases.

**Results:**

From datasets including ERPs from the mid-frontal, mid-parietal, right frontal, and central scalp areas, we found 82% of agreement between DSM and biological-based classifications. The kappa index between DSM and behavioral/psychological/neurophysiological data was 0.75, which is regarded as a “substantial level of agreement”.

**Discussion:**

The CLV is a useful method for multivariate analysis of datasets with much less subjects than variables. In this study, a correlation is found between the biological-based classifier and the DSM outputs for the classification of subjects as either ADHD or not. This result suggests that DSM clinically describes a biological condition, supporting its validity for ADHD diagnostics.

## Introduction

The validity of the Diagnostic and Statistical Manual of Mental Disorders—DSM (American Psychiatric Association, 1994–2013) (American Psychiatric Association, 1994; American Psychiatric Association, 2013) is usually questioned due to its subjective criteria and to the absence of objective tests to define nosological entities (Insel, 2018; Wakefield, 2015). Therefore, the lack of biological correlates makes room for criticism and raises doubts about the nosological criteria itself (Wakefield, 2015). This is particularly important in controversial mental diseases such as the Attentional Deficit/Hyperactivity Disorder—ADHD (Rafalovich, 2005; Strauss, 2018). Similar to some other disorders, ADHD diagnosis using DSM scores is based on the subjective evaluation of the expression of behavioral dimensions. Thus, DSM has a taxonomy of categories that are defined by dimensional phenomena based on a variation from normality (Maser & Akiskal, 2002; Kirmayer & Crafa, 2014). Correlations between biological alterations and ADHD are still not enough to support the diagnosis (Kirmayer & Crafa, 2014; Furman, 2008).

A mental disorder could potentially be explained by a set of interdependent, objectively measurable biological dimensions (such as EEG- and Event-related potentials, and psychometric measures such as reaction time, etc.), since mental phenomenology stem from biological systems. However, it is still not possible to perform any reductionist analysis that could unambiguously uncover the cerebral mechanisms of mental disorders or reveal any biomarkers that determine any diagnosis.

Mental disorders are multidimensional complex entities, with non-linear relationships among their biological processes (Kirmayer & Crafa, 2014; Freitas-Silva & Ortega, 2016) rather than being defined by a single distinct biomarker such as an antibody or aberrant protein (Scarr et al., 2015). Thus, a complex nosological entity, such as ADHD, may be explained by a large set of different quantitative variables of biological dimensions.

The objective of this study was to evaluate the accuracy of DSM based on the correspondence between clinical classification and classification using multidimensional biological measures from neurophysiological and behavioral data. Thus, we have assumed that these biological data contain patterns that can explain the nosological entity called ADHD. It does not matter whether we know which these patterns are or not as long as they are really embedded in these objective measures. Multivariate analysis can empirically find these patterns that synthetize this complexity. These informative patterns are treated as latent variables (LVs).

From this standpoint, multivariate analysis techniques have been applied to electroencephalographic or biochemical data since the 1970’ in order to develop high-sensitivity and accuracy models for the diagnosis of mental disorders (Bochkarev et al., 1987; Lazarev, 2006; Lazarev et al., 1990; Monakhov et al., 1979; Schwarz et al., 2010). However, in order to explain these complex mental conditions in terms of biological correlates, a substantially larger sample of subjects than those set of variables is required. Thus, high-dimensional datasets from relatively few observations or subjects usually appear to be statistically questionable and hamper unambiguous interpretation. In order to tackle this problem (sometimes called the curse of the dimensionality), approaches based on feature selection in a supervised context or on feature extraction in explorative data analysis (for instance, Principal Components Analysis, Canonical Correlation Analysis) have been investigated. Particularly, a strategy of unsupervised “clustering of variables around LVs” (CLV) has been used. CLV approach allows for extracting synthetic/latent variables through cluster analyses (Vigneau & Qannari, 2003; Vigneau, Chen & Qannari, 2015). These few LVs hold the dataset informativeness providing an integral view of the data, which can be used in a subsequent classification methodology (e.g., inputs for a k-means clustering algorithm). This was the first attempt to apply the CLV method to a large psychophysiological dataset. This method helps to solve an important methodological problem of multidimensional approaches in clinical research when recruiting large groups of patients is difficult.

In the present study, our aim was to deal with a small sample of subjects with well-controlled confounding variables and bias. A large number of characteristics have been collected from typically developing boys (TD) and boys diagnosed with ADHD using the DSM-IV-TR criteria. There were three groups of data: (1) behavioral (related to reaction time –RT), (2) neurophysiological (event-related potentials (ERPs), including late cognitive component P3 or P300 (Hruby & Marsalek, 2003; Barry, Johnstone & Clarke, 2003), both obtained while subjects performed the Attention Network Test (ANT) (Fan et al., 2002; Kratz et al., 2011)), and (3) psychological (from WISC-III). The relationships between ADHD manifestations, evaluated using DSM-IV-TR, and the objective characteristics obtained by the above-mentioned experimental measurements were here analyzed to discuss the taxonomic (i.e., classificatory) aspects of ADHD diagnosis using DSM, compared with a data-driven statistical approach.

This study does not address controversial issues, e.g., if the ADHD phenomenology actually constitutes a mental disorder or not. The present study seeks to infer about the accuracy of the DSM as a tool to identify this phenomenology, which could be important for further discussions about the validity of this manual for ADHD diagnostics.

## Materials & Methods

### Design and subject selection

This transversal and exploratory study was conducted in accordance with the Declaration of Helsinki and approved by the Ethics Committee of the National Institute of Women, Children, and Adolescents Health Fernandes Figueira. Before the first recruitment, the project was registered at plataformabrasil.saude.gov.br, under the number CAAE 08340212.5.0000.5269. All the participants provided their oral consent in the presence of their caregivers, who provided written informed consent after receiving a complete description of the study.

Sixty boys, aged 10–13 years, were included according to DSM-IV-TR: 35 with ADHD (coded from T001 to T035) and 25 typically developing (TD, coded from C001 to C025). The exclusion criteria were: (1) history of chronic diseases, and any suspicion of psychiatric disorders other than ADHD (psychosis, affective, obsessive-compulsive and tic disorders, phobic and post-traumatic stress conditions, anorexia, bulimia, encopresis, or enuresis) as screened by K-SADS-PL (Matuschek et al., 2016); (2) use of any psychotropic medicines for at least 30 days; (3) estimated Intelligence Quocient (I.Q.) equal or lower than 80; and (4) less than 6 h of regular sleep and (5) report of somnolence before the ANT testing.

The following were considered as confounding variables: years in school, monthly family income (in Brazilian Reals), sleep hours the night before, and mean weekly time which the boy dedicated to computer activities and videogames (ranked as follows: 1, less than 2h/week; 2, 2–4 h; 3, 5–8 h; 9–14 h, more than 15 h/week), as well as age.

After applying the exclusion criteria and performing the behavioral analysis of accuracy (AC) and speed-accuracy tradeoff (see below), 19 ADHD and 20 TD boys remained in the sample for analysis.

### Clinical and psychological examination

Each subject was evaluated using a structured interview where his or her caregivers were shown the DSM-IV-TR criteria and were instructed to point out carefully whether or not each specific criterion was an exact characteristic of their child’s behavior. If there was any doubt about or hesitation concerning any item, it was disregarded. Thus, subjects were classified as typically developing (TD) (comprising the control group) or ADHD in accordance with the DSM-IV-TR.

I.Q. was estimated by Block Design and Vocabulary subtests from the Wechsler Intelligence Scale for Children, WISC-III (Wechsler, 1991; Mello et al., 2011). The Arithmetic and Digit Spam WISC subtests were also performed and their scores were included in the pool of variables used in the multivariate analysis.

### Experimental procedures

The ANT version adapted for children was used (Kratz et al., 2011; Abramov et al., 2017). It was performed by an inhouse software. A forced, two-choice test was performed. In a dim examination room, the subject sat comfortably in front of the rectangular LED monitor with cyan background (size of 25° ×18°; mean luminance 40–50 cd/m^2^), at 50 cm distance between nasion and screen), fitting his/her glance on the central black cross (1.4° ×1.4°). He was instructed to observe the horizontal orientation of a yellow fish as target stimulus (1.7° ×1.1°), appearing for 350 ms, which was (or not) preceded by a cue signal (red star, 1.4°) with onset of 1,650 ms before the target and 150 ms duration. The target appeared above or below the fixation cross (distance of 3,5°), always flanked by two distractors at each side, which were identical yellow fishes oriented towards the same (congruent) or opposite (incongruent) direction of the target. The flank distractors appeared 100 ms before the target (according to Kratz et al., 2011). The random interval between the trials was 1–2 s. There were three equiprobable cue conditions corresponding to this signal’s position or to its non-appearance: (1) at the subsequent upper or lower position of the target—spatial cue condition; (2) at the central fixation point—neutral cue condition; or (3) no cue condition. The subject had to promptly press the left or right arrow key on the keyboard, according to the horizontal orientation of the target. The test was organized in 8 test blocks, with 24 trials each, and one preceding training block. RT and accuracy (AC, rate of hits) were recorded.

Subjects with AC lower than 70% and speed-accuracy tradeoff (estimated as AC × RT) lower than the mean sample value minus two standard deviations were excluded.

### EEG acquisition

**Figure 1:**
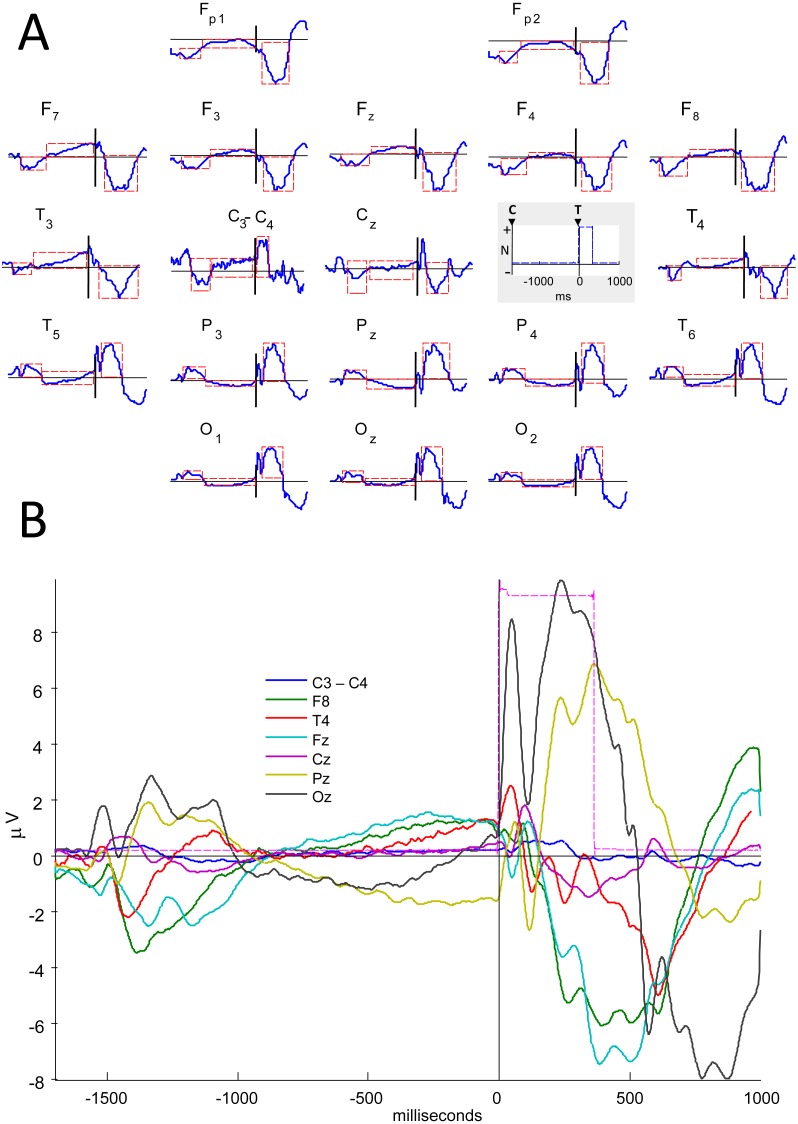
Electrical potentials related to Attention Network Test. (A) Cue- and target-related potentials and variation in interstimulus voltage during Attention Network Test performance (averaged over all ANT conditions and all subjects). Overview of the ERPs for all channels, including ‘C3 minus C4’ (normalized amplitudes, µV), with waves of interest (in dashed red rectangles) to calculate the peak amplitudes/latencies. In the grey box, C and T stand for cue and target onsets, and the trigger signal is identified by blue dashed lines, which mark the target time. Amplitudes are normalized (N), with polarities indicated. Scalp areas are marked with letters with even (right), odd (left) or z (midline) indices: occipital –O, parietal –P, central –C, frontal –F, frontopolar –Fp, mid-temporal –T, posterior temporal –T5 and T6, and anterior temporal –F7 and F8. (B) ERPs for selected channels, in different colors, showing wave amplitudes (µV). The reference was C3+C4.

During the ANT, the subject’s EEG was recorded using a Nihon Kohden NK1200 EEG System at 20 scalp points according to the International 10/20 System (Fig. 1A), with reference at the lateral central leads (linked C3 and C4, the physical reference of the System). Impedance was below 10 kΩ, sampling rate was 1,000 Hz with a 16-bit resolution, and the filters were as follows: high-pass 0.5 Hz, low-pass 100 Hz, and notch 60 Hz. Grossy muscular and movement artifacts (mainly those of higher amplitude) were manually removed under visual inspection of the signal. The blinking artifacts were attenuated by FilterBlink method (Abramov et al., 2018). The ANT was performed and presented using Psychotoolbox (psychtoolbox-3.org).

## Data Analysis

Regarding the comparison tests between ADHD group and TD group of subjects (Table 1), we performed t-tests based on robust estimators of location (modified one-step M-estimator, MOM) and bootstrap procedure. This test procedure is available in the WRS2 R package (pb2gen function) (Wilcox & Rousselet, 2017). To evaluate the effect of reclassification on the ERPs, we compared the peak amplitudes of the target-related parietal P3 wave between Control and ADHD groups, formed either by DSM classification or by the biological-based classifier.

Several behavioral variables were gathered from the ANT for each subject: mean AC of task performance, i.e., the percentage of correct responses; mean RT and its standard deviation, called intra-individual variation of RT (IVRT), for each cue and target condition. Additionally, data included the ANT scores of the subjects: alerting, orienting, and conflict resolution, mean RT for correct and incorrect responses, and learning rates (the mean scores of the first ANT trial block divided by those of the last experimental block evaluated for RTs and IVRTs).

The peak amplitude, peak latency, and mean amplitude of the ERPs were estimated inside three time intervals of interest (see dashed rectangles in Fig. 1A and their measures in Table S1) for each subject, in each cue and target condition and in each EEG derivation (except for C3 and C4). These three time intervals included cue- and target-related late potentials, and the voltage variation between these two ERPs. The same ERP parameters were calculated for the difference between signals of the two central channels (C3 minus C4) instead of estimating the absolute signal values in each of these leads, observing the EEG reference used here (C3+C4).

Thus, the total number of variables of interest (VOI) was 774: four scores obtained from the above-mentioned WISC-III subtests, 29 behavioral variables obtained from ANT, and 741 neurophysiological variables (39 for each of the 18 channels and C3-C4).

The neurophysiological variables obtained represent topographical parameters of cerebral activity, while the variables from ANT and WISC provide the integral characteristics of the subject. For the analysis, the activity in certain brain regions of the subjects performing ANT (but not all) was assumed to best correlate with ADHD phenomenology (even EEG cannot reveal which areas are these ones). For this reason, 40 different channel sets (Table 2) were used for classifications, in an attempt to find the best agreement with DSM. The dataset with variables used in the multivariate analysis is available in the (Dataset S1). Wave data is disposed in the Figshare repository at DOI: 10.6084/m9.figshare.7914422.

**Table 1:** DSM scores and Confounding Variables. Videogame and computer handling were scored as: 1 = less than 2h/week; 2 = 2–4h/wk, 3 = 5–8 h/wk, 4 = 8–15 h/wk, 5 = more than 15 h/wk. Inference by Student’s *t*-test based robust estimator of location (modified one-step, MOM) and bootstrap procedure.

	Typically Developing (*n* = 20)	ADHD (*n* = 19)	stat	*p* value
	MOM estimator for location	MOM estimator for location		
DSM scores				
**Inattention**	**2.40**	**7.21**	**−4.81**	**<0.001**
Hyperactive + Impulsive	2.37	4.37	−2.00	0.09
**Total**	**5.00**	**11.28**	**−6.27**	**<0.001**
Confounding variables				
Age (years)	11.11	11.52	−0.41	0.314
**Estimated I.Q.**	**109.35**	**97.37**	**11.98**	**0.010**
Hours of sleep (last night)	7.53	8.00	−0.47	0.357
Videogame	4.00	2.89	1.10	0.113
Computer handling	3.93	2.95	0.99	0.317
Years in school	6.02	6.10	−0.05	0.646
Family income (monthly)	4,006.00	2,741.00	1,265.00	0.437

**Table 2:** Agreement between DSM and Biological-based classifier for Trainning and Test subsets. Channel sets according to the International 10–20 system for EEG leads; C3–C4, the channel of difference between two leads. In bold, higher DSM agreement. ERPs = event-related potentials; LV = latent variables. Modal value = the value the most often observed.

Channel sets	train set (31/39=79%)		Test set (8/39=21%)	
	N. LVs (modal value)	Mean % of agreement	Mean % of agreement	Number of variables
33 [** C3-C4 F8 F4 Fz Pz]**	1 (60%)	80,1%	76,4%	*130*
37 [C3-C4 F8 T4 F4 Fz Pz]	1 (56%)	80,2%	74,9%	*156*
13 [ C3-C4 Fz Pz]	1 (55%)	79,9%	74,6%	*78*
12 [ C3-C4 F8 T4 Fz Pz]	1 (48%)	77,6%	73,6%	*130*
34 [F8 F4 Fz Pz]	1 (67%)	76,5%	72,3%	*104*
39 [Fz Pz]	2 (60%)	76,2%	71,9%	*52*
8 [ C3-C4 Fz Cz Pz]	1 (67%)	71,3%	71,5%	*104*
17 [F7 F3 C3-C4 F8 F4 Fz Pz]	3 (31%)	76,4%	70,9%	*182*
21 [F7 Fp1 F3 C3-C4 F8 Fp2 F4 Fz Pz]	2 (26%), 3 (24%)	74,2%	70,9%	*234*
31 [F7 F3 C3-C4 Fz Pz]	2 (23%)	76,4%	70,3%	*130*
15 [F7 C3-C4 F8 Fz Pz]	2 (38%)	75,4%	70,0%	*130*
19 [F7 F3 F8 T4 F4 Fz Pz]	3 (41%)	74,7%	69,7%	*182*
40 [Fz Cz Pz]	1 (33%)	71,4%	69,5%	*78*
36 [F7 T3 F3 C3-C4 P3 Fz Pz]	1 (31%)	71,9%	68,6%	*182*
22 [F7 Fp1 F3 F8 Fp2 F4 Fz Pz]	1 (28%), 2 (26%)	74,2%	68,5%	*208*
16 [F7 F3 F8 F4 Fz Pz]	3 (38%)	76,9%	68,4%	*156*
38 [ F7 T3 F3 C3-C4 Fz Pz]	2 (32%)	73,4%	68,1%	*156*
29 [C3-C4 F8 F4 P4 Fz Pz]	2 (48%)	76,0%	67,9%	*156*
32 [F7 F3 Fz Pz]	2 (39%)	73,0%	67,8%	*104*
26 [F7 F3 P3 F8 F4 P4 Fz Pz]	2 (40%)	71,5%	67,6%	*208*
11 [ F7 T3 C3-C4 Fz Pz]	2 (34%)	74,7%	67,5%	*130*
35 [C3-C4 F8 T4 F4 P4 Fz Pz]	1 (37%), 2 (30%)	74,6%	67,4%	*182*
27 [F7 F3 C3-C4 P3 Fz Pz]	1 (22%), 2 (18%)	70,5%	66,6%	*156*
23 [F7 Fp1 F3 C3-C4 F8 Fp2 F4 Fz]	1 (27%), 2 (25%)	72,3%	65,4%	*208*
30 [F8 F4 P4 Fz Pz]	1 (26%), 2 (19%)	72,6%	65,3%	*130*
24 [F7 Fp1 F3 F8 Fp2 F4 Fz]	1 (26%), 2 (24%)	72,6%	65,2%	*182*
28 [F7 F3 P3 Fz Pz]	1 (36%)	70,4%	65,0%	*130*
25 [F7 F3 C3-C4 P3 F8 F4 P4 Fz Pz]	1 (31%), 2 (35%)	72,4%	64,9%	*234*
18 [F7 F3 F8 F4 Fz]	3 (27%)	70,5%	64,8%	*130*
9 [F7 T3 T5 C3-C4 Fz Pz]	2 (46%)	68,1%	63,3%	*156*
20 [F7 F3 C3-C4 F8 F4 Fz]	1 (34%)	72,2%	62,6%	*156*
2 [Fz Cz Pz Oz]	1 (24%), 2 (23%)	66,7%	61,0%	*104*
4 [C3-C4 F8 T4 T6 Fp2 F4 P4 O2 Fz Pz]	1 (73%)	62,9%	60,9%	*260*
10 [C3-C4 F8 T4 T6 Fz Pz]	2 (37%)	68,6%	59,9%	*156*
5 [F7 T3 T5 F8 T4 T6 Fz Cz Pz]	2 (49%)	65,3%	59,8%	*234*
6 [F7 T3 T5 C3-C4 F8 T4 T6 Fz Cz Pz]	2 (32%) 3 (20%)	66,4%	59,1%	*260*
3 [F7 T3 T5 Fp1 F3 C3-C4 P3 O1 Fz Pz]	2 (28%), 3 (35%)	66,6%	56,1%	*260*
7 [F7 T3 T5 Fp1 C3-C4 F8 T4 T6 Fp2 Fz Cz Pz]	2 (38%)	65,9%	56,0%	*312*
14 [F7 T3 T5 C3-C4 F8 T4 T6 Fz Pz]	2 (25%), 3 (23%)	64,7%	55,5%	*234*
1 [All Channels]	2 (37%), 3 (23%)	66,4%	55,3%	*494*
ANT+WISC	1 (75%)	66,0%	65,0%	*33*
WISC+ANT+[** C3-C4 F8 F4 Fz Pz]**	1 (78%)	80,7%	76,4%	*152*

The data from each investigated set of EEG channels were analyzed either with or without behavioral (ANT) and psychological (WISC) variables. They were submitted to CLV, an unsupervised method for multivariate analysis (Vigneau & Qannari, 2003; Vigneau, Chen & Qannari, 2015). The aim was to reduce the number of dimensions in the variables space by extracting latent variables (LVs), each of them being associated with a cluster of the variables of interest (VOI). This clustering approach is based on correlation similarity indices between the variables and aims to identify directional clusters of variables. As it is, each extracted LV, associated with a specific cluster of variables, is defined as the first (standardized) principal component of the variables assigned to that cluster (Vigneau & Qannari, 2003; Vigneau, Chen & Qannari, 2015). There is detailed information about CLV methodology in Supplemental Method S1. In our study, the clusters of variables around one to six LVs were systematically considered and the best number of LVs was determined according to the classification of subjects (which is detailed in the next paragraphs).

Our ultimate purpose was to reclassify the subjects into two groups according to the objective behavioral, psychological, and neurophysiological data, all of which were summarized around the extracted LVs. A k-means clustering method (Gore, 2000) was performed on the subjects using the retained LVs. Each reclassification obtained (named “output”) was checked for agreement compared to the classification of subjects using the DSM-IV-TR (called “DSM agreement”). The probability that agreement with DSM could be observed under randomness was checked using the Chi-Square test for categorical variables with one degree of freedom. The kappa index (Landis & Koch, 1977) was also evaluated to estimate the level of agreement with DSM (Supplemental Method S2).

In order to determine whether reclassification outputs are robust against a variation of the subject’s sample, a cross-validation approach has been undertaken. The objective was to better assess the performance of our numerical processing workflow by repeatedly simulating a training sample set, on which the statistical model is built, and an independent test set, on which the reclassification performance is finally evaluated. In practice, the whole sample of the 39 subjects is split into a training set of 31 subjects (about 80%) and a test set of 8 subjects (about 20%). The selection of the test set observations is performed so as to have the same proportion of ADHD and TD subjects (by DSM classification) in the test set as in the whole set. The CLV method is applied on the training set, and one to six LVs are considered for k-means clustering into two clusters. The number of LVs to be retained is based on the percentage of agreement with the data driven partition and the DSM diagnostic. The last step is to predict group membership of the test set subjects, who play the role of independent observations, using statistical modelling.

This cross-validation procedure is repeated a large number of times. Herein we performed 100 repetitions. The mean level of agreement in reclassification across all the test sets is the performance indicator we considered for identifying the combination of EEG channels providing the best outputs. See Supplemental Method S3 for a graphic representation of the cross-validation method.

Considering the best choice of VOI variables, a sensitivity and specificity analysis for the ADHD DSM criteria is suggested.

## Results

### Evaluating DSM and confounding variables

For descriptive purposes, we compared DSM scores of two samples, with subjects individually classified as ADHD or TD according to the DSM: as expected, Control and ADHD groups showed significant differences regarding DSM-IV-TR criteria for attention and total scores (*p* < 0.001, ADHD > TD scores). The hyperactivy+impulsivity scores are not different between groups (*p* = 0.09). Among variables considered as confounders, the I.Q. scores in ADHD group were lower than in TD boys (*p* = 0.010) although they were always higher than 80. No other variables were significantly different between the two groups. See these results in Table 1.

### Overview of event-related potentials

**Figure 2:**
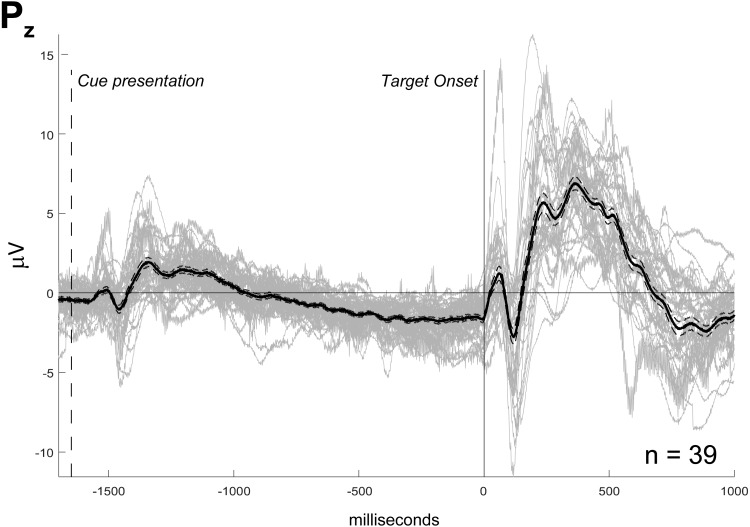
ERP sample from Pz channel. The individual waves (*n* = 39, in gray) are superimposed to their mean value (black thick line) and their standard error of mean (black thin dashed line). Amplitude in microvolts (µV). Markers indicating target onset (solid line) and cue presentation (dashed line). Waves related to all ANT conditions indistinctly averaged.

Cue and target-related potentials with late peak latency (>200 ms, corresponding to parietal P3) were observed in all channels. Peak amplitude varied from the maximum value of 18 µV for the frontopolar target ERP to the minimum value of 1 µV for the difference between C3 and C4 (Fig. 1B). In Fig. 2, each subject’s waves are plotted to evaluate sample consistency of ERPs from the mid-parietal region (Pz). In Fig. S1 there is the same information for all channels. We can observe apparent differences between waves for each ANT condition, which are not statistically tested in this work (Fig. S2).

### Reclassification of subjects

According to a preliminary data analysis, it turns out that the mean amplitude was highly correlated with the peak amplitude measurement. The mean amplitude was thus discarded and only peak amplitude and peak latency measures were subsequently considered.

For all the 40 channel sets, values of agreement with DSM (Table 2) varied from 66.4% to 80.1% considering the results observed on the training sets, and varied from 55.3% to 76.4% based on the test set results. These results were only observed using the ERP channels. If behavioral variables from ANT and WISC scores were used conjointly with the channel sets, we obtained more or less the same performance than without these additional variables (data not shown).

Based on the results of the test sets, the worst mean agreement level between DSM and k-means reclassification was 55.3% when all the ERP channels were involved. The best mean agreement level with DSM was 76.4% for [C3-C4, F8, F4, Fz, Pz] channel set (set 33). Two other channel sets showed a good performance (Table 2). Those ones were [C3-C4, F8, F4, T4, Fz, Pz] channel set (set 37) and [C3-C4, Fz, Pz] channel set (set 13). In these three cases, the number of channels considered is quite small (3, 5 or 6 over 19) and the difference between C3 and C4, as well Fz and Pz, is involved.

The set of channels [C3-C4, F8, F4, Fz, Pz] was chosen as the model, with only one LV, and has been rebuilt using the now 39 available subjects. Globally, the output shows that seven subjects were miss-reclassified: five controls (C005, C009, C012, C020, C023) to the ADHD group, and two ADHD subjects (T010, T026) to the control group. There was an accuracy of 82.05% (32 subjects, *χ*^2^ = 7.20, *p* = 0.004). The estimated specificity and sensitivity of the DSM-IVTR regarding these ERP variables were 75.00% and 89.47%, respectively. This accuracy corresponds to the kappa index of 0.75, i.e., a substantial strength of agreement (Landis & Koch, 1977).

**Figure 3:**
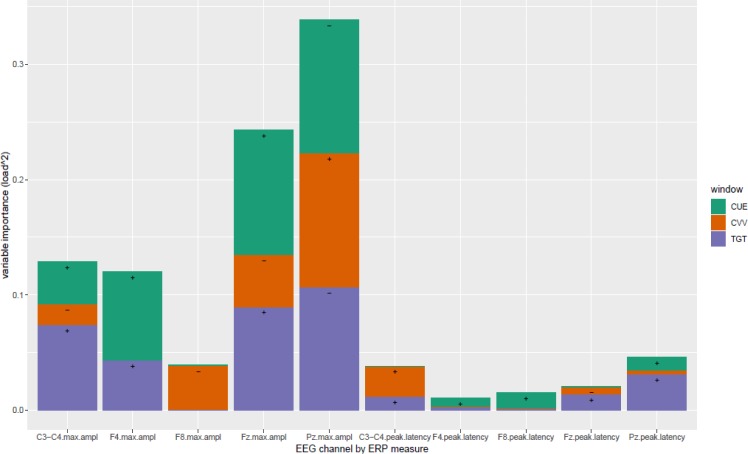
Variable Importance in the channel set with larger agreement. Importance is represented in terms of Loading ^2^ comprising the first latent variable (more representative to larger agreement) in [Fz, Pz, C3-C4, F4, F8] channel setting, by CLV method. Load directionalities are indicated by (+) or (−); max.ampl = peak amplitude, CUE = cue signal, CVV = contingent voltage variation, TGT = target signal. See figure 1 for channel topography.

Moreover, the loadings of the VOI associated with this LV should be considered in order to better understand what information is actually involved in the classification. More specifically, as the squared values of loadings total one, each squared loading may be considered as reflecting the importance of the corresponding variable. Figure 3 shows these importance indices by channel, as a function of the type of window presented and the type of measurement collected. It is evident that the most important type of measurement is maximum amplitude, and the peak latency measurement is not quite informative to retrieve the DSM classification. Observations on Fz and Pz channels (with opposite values of loading as shown with the sign annotated in the bar in Fig. 3) appeared to be the most important, specifically for Cue and Target-related ERPs. The loadings associated with C3–C4 and F4 channels are in the same direction as Fz loadings.

**Figure 4:**
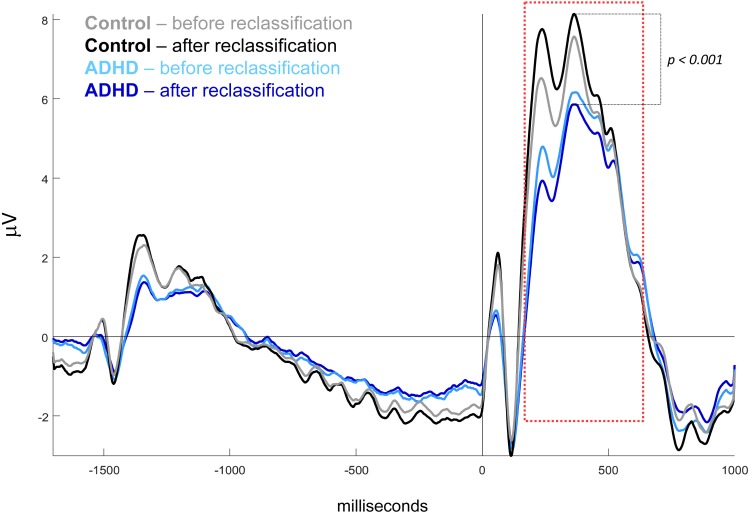
Mid-parietal ERPs before and after subject’s reclassification. ERP waveshapes after reclassification, when miss-classified subjects by DSM are relocated by biological-based classifier. Sample means for ADHD before (light blue), ADHD after (dark blue), Control before (gray), Control after (black) reclassification. Amplitude in microvolts, target onset at 0 ms. Waves related to all ANT conditions indistinctly averaged.

After reclassification of subjects by the above biologically based classifier, the mid-parietal (Pz) P300 wave showed higher difference between the two new groups than between those based on the DSM classification. In the former case, this difference was very highly significant: 10.64 in controls (MOM estimator) vs. 7.43 in ADHD (Test statistic = 3.22, *p* < 0.0001), while in the latter, it was not significant: 10.13 vs. 8.13, respectively for control and ADHD groups (Test statistic = 1.99, *p* = 0.103) (Fig. 4).

## Discussion

Our study tackled the accuracy and biological adequacy of examining attention deficit/hyperactivity disorder using the DSM manual, with reference to classifier variables based on objective variables of biological nature. This classifier has comprised a new synthetic dimensional reduction method, the CLV (Vigneau & Qannari, 2003; Vigneau, Chen & Qannari, 2015), and a traditional clustering algorithm (k-means). Comparing the classifications, the DSM seemed to be justified for ADHD diagnosis with “substantial strength of agreement”. A larger source of behavioral and neurophysiological data related to ADHD phenomenology was found in the ANT, since this test is suitable to access the three dimensions of attention in the same setting (Fan et al., 2002; Petersen & Posner, 2012).

In order to classify subjects as TD or ADHD according to DSM criteria, caregivers were asked which criteria were well-defined characteristics of their children. Other assessment methodologies should also be used along with DSM for ADHD diagnosis both in scientific research and in clinical practice. Moreover, the manual should not be applied as a questionnaire in clinical practice. However, in this case, the object of evaluation is the DSM itself; it was thus the only clinical diagnostic tool used for the classification of subjects as either ADHD or not-ADHD. It was the most objective and controlled way to obtain the scores without interference from any other clinical impression. Supporting the present methodology, the ASRS (the WHO questionnaire for adult ADHD screening) was self-administered (Kessler et al., 2005).

Although this study was not focused on neural mechanisms of ADHD, it seems to be important to tackle this aspect briefly to show that there is real neurophysiological basis under our models. The selected neurophysiological variables with which DSM best agrees were topographically asymmetrical and represented the binded information from ERPs mainly in cue- and target-related late components from mid-frontal (Fz), mid-parietal (Pz), right frontal (F4), and right anterior temporal (F8) sites. The P3 wave, a late potential related to cognitive tasks (Hruby & Marsalek, 2003) is particularly sensitive to ADHD conditions (Barry, Johnstone & Clarke, 2003; Kratz et al., 2011), which justifies the importance of cue- and target-related late potentials in classification, mainly in the mid-parietal region where this wave is most pronounced. In a recent study, we found an ERP asymmetry (from 45 to 290 ms after target onset) in the ‘C3 minus C4’ channel, which was shown to correlate with ADHD phenomenology (Abramov et al., 2016). Several studies have shown brain asymmetries related to ADHD phenomenology, especially in the frontal-striatal network (Barkley, 1997; Barkley, 2006; Langleben et al., 2001; Dang et al., 2016). Asymmetrical topographic patterns were shown in our previous studies by spectral and coherence analyses of the resting EEG, with signs of relative inactivation of the frontal and left temporal areas, in accordance with their importance for voluntary attention, which is impaired in ADHD subjects (Lazarev et al., 2016).

Due to the correlation with the pattern of variables compatible with the current biological models for ADHD, the present result suggests that DSM-IV-TR is actually a biologically justified tool for ADHD diagnosis, as it was applied here. Although a Kappa index of 0.75 should be considered with reservations for some clinical settings, for mental disorders, a clinical method such as DSM showing this level of agreement with biological characteristics should be considered as particularly relevant. First, we must consider the inherent complexity of mental disorders that have no validated biomarkers. This complexity reaches its maximum when we deal with dimensional clinical phenomenology, whose symptoms are simply, sometimes extreme, variations in the expression of normal behavior, as is the case of ADHD. How many different measures are necessary to cluster these conditions? Furthermore, the ADHD has been regarded as a heterogeneous condition with inattentive, hyperactive, and combined subtypes, which could be the answer for this level of agreement since these subtypes were not intended for reclassification. The overlay of three discrete subtypes could lead to a flawed reclassification.

The only psychiatric tool for diagnosis and intervention is still phenomenological examination, which must be systematically (although qualitatively and subjectively) performed based on judgement about whether the subject meets the criteria from manuals such as the DSM. Thus, it is reasonable to regard that the information embedded in the objective measures from the EEG and behavior in ANT, even distorted by all those nonlinearities, is more reliable a priori for classifying subjects than the DSM, which is a subjective assessment.

The small sample size can naturally raise doubts. However, this sample was designed to be very consistent and homogeneous. Any subject with the slightest suspicion of comorbidity was excluded. Potentially confounding factors commonly ignored were monitored (such as computer and videogames that could lead to the development of specific skills that would interfere with test performance and socioeconomic niche that could determine psychological development). Therefore, the sample should be regarded as well representative of ADHD. On the other hand, in order to classify the subjects according to their mental condition (a multidimensional phenomenological complex) it is necessary to gather a considerable number of objective variables (as many as possible). Since it did not seem feasible to collect a sample with thousands of subjects evaluating hundreds of variables in an experiment with optimal control of confounders and bias, the dataset had to have a much smaller number of observations than variables. A small but strictly controlled and well-exploited sample was considered preferable, even in conditions of high prevalence such as the ADHD. Multivariate statistical modeling, such as Linear Regression, Discriminant Analysis, or Factor Analysis, are well known as unsuitable for this situation. Thus, in order to tackle a frequently encountered methodological problem, often referred to as the curse of the dimensionality, an explorative data analysis suitable for small samples was adopted to identify a small number of synthetic latent variables. Instead of the Principal Component Analysis, which does not always provide easily interpretable LVs (the principal components), an approach with clustering of variables was chosen to reduce data dimensionality to a small number of new LVs. The CLV method (Vigneau & Qannari, 2003; Vigneau, Chen & Qannari, 2015) has already been applied to a wide range of research domains among which are chemometrics (Vigneau et al., 2005b), sensory analysis (Vigneau et al., 2005a), image analysis (Legland & Beaugrand, 2013), and analysis scorecards in the health sector (Lovaglio, 2011). The results seemed promising in optimizing the experimental designs of clinical studies in which it is difficult to recruit large groups of patients.

A major limitation of this study is the impossibility of testing all possible combinations among variables. Since the consistency of our paradigm lies on the fact that no biological / behavioral assumptions were considered in the grouping of variables, it is necessary to test them randomly grouped. However, there are more than one million possible combinations of EEG channels. The number of combinations grows exponentially if the other variables are included individually. For computational feasibility, sets of variables were arbitrarily chosen for analysis. Considering the eighty sets studied, it can be assumed that DSM has at least a ”substantial level of agreement” with biological determinants. Perhaps some untested set of variables may manifest higher agreement with the DSM, but the results found already satisfy our objective of testing the accuracy of the DSM criteria for ADHD, discussing about its adequacy for it.

In the ANT paradigm used here (Kratz et al., 2011), the flank-target offset of 100 ms compromises the evaluation of target-related early potentials. The first flank-related early potential appears just at the target onset, and the first target-related wave is superimposed to other flank-related elements. However, this did not compromised our results, since we did consider only measures from late ERP components, such as contingent voltage variation, because several authors clearly point to a correlation between alterations in cognitive conditions and these components, in particular, the P3 wave (Hruby & Marsalek, 2003; Barry, Johnstone & Clarke, 2003). In the previous ERP study using ANT, contingent voltage variation, cue and target-related P3 waves were modulated by ADHD condition (Kratz et al., 2011).

The estimated I.Q. scores were different between the groups diagnosed using DSM-IV-TR, but this was not regarded as bias. Literature has shown that intelligence tests are sensitive to ADHD, and scores of TD subjects are generally higher (Mackenzie & Wonders, 2016).

Even taking into account the above restrictions, there is no doubt that the data obtained seem to be quite consistent.

## Conclusions

Using the CLV method, suitable for samples with a much smaller number of cases than the number of variables analyzed, we conclude that the DSM criteria “A” for ADHD diagnosis have a substantial (nearly 80%) correlation with the biological dimension, especially with the neurofunctional responses related to attentional networks of Posner (Fan et al., 2002). Thus, DSM, as applied here, could be considered as a biologically adequate and accurate clinical tool for ADHD diagnostics.

##  Supplemental Information

10.7717/peerj.7074/supp-1 Supplemental Information 1 Clustering around Latent Variables (CLV) method Detailed mathematical description of CLV method.

10.7717/peerj.7074/supp-2 Supplemental Information 2 Calculating the Kappa Index Inference of the magnitude of agreement between two categorical data (in this case, subject classification according to DSM and to biological/behavioral variables in the dataset).

10.7717/peerj.7074/supp-3 Supplemental Information 3 Description of Cross-validation methodology For emulate independent samples –see main text for details.

10.7717/peerj.7074/supp-4 Table S1 Time intervals of Interest Windows holding Cue-related potentials (CUE), contingent voltage variation (CVV), and Target-related potentials (TGT) for each channel (ms) to calculate peak amplitude, peak latency, and mean amplitude. Zero ms refers to Target onset (see Fig. 1).

10.7717/peerj.7074/supp-5 Dataset S1 DSM scores, confounding variables and variables of interest In the first sheet: inattention, hyperactive+impulsive, and total scores for ADHD according to DSM-IV-TR; second sheet: all data concerning (1) scores of the WISC-III subtests, (2) ANT performance (AC: accuracy, RT: reaction time and IVRT: intraindividual variation in reaction time, which is the standard deviation of the subject’s RT) for each experimental condition (Allcon: mean value of all conditions, NtCue: neutral cue condition, SpCue: spatial cue condition, ConTg: congruent target condition, IncTg: incongruent target condition) and attentional network (alerting, orienting, and executive) plus learning indexes (IVRT or RT at the last ANT block/first ANT block), and (3) the neurophysiological measures (peak amplitudes and latencies, and mean amplitudes inside each time interval of interest in S1) for the EEG channel set.

10.7717/peerj.7074/supp-6 Figure S1 Samples of ERPs by channel Individual waves from the sample for each channel (all conditions), by page. The waves (gray) are superimposed to their mean values (black thick line) and their standard error of mean (black thin dotted line). Amplitude in microvolts. Waves related to all ANT conditions indistinctly.

10.7717/peerj.7074/supp-7 Figure S2 Waves by ANT condition Sample means (*n* = 39) of No Cue (blue), Neutral Cue (black) and Spatial Cue (red) -related potentials are superimposed in (A), and Congruent (blue) and incongruent (black) -related target conditions are superimposed in (B). See caption of figure 1 for more details.

## Additional Information and Declarations

### Data Availability

The following information was supplied regarding data availability: The raw data is available in the Supplemental File and the raw wave data (ERPs by subjects) is available at FigShare: Abramov, Dimitri (2019): Estimating Biological accuracy of DSM for Attention Deficit/Hyperactivity Disorder based on Multivariate Analysis for Small Samples. figshare. Dataset. DOI: 10.6084/m9.figshare.7914422.v1.
