# Supplementary material for: Estimating biological accuracy of DSM for attention deficit/hyperactivity disorder based on multivariate analysis for small samples
Source: PeerJ. 2019 Jun 12;7:e7074. doi: 10.7717/peerj.7074 (PMC6571005; doi:10.7717/peerj.7074)
Supplement: Supplemental Information 1 — Detailed mathematical description of CLV method. [file peerj-07-7074-s001.pdf]

Let us consider a matrix **X** of *p* numerical variables observed on *n* observations.Two objectives for the clustering of the variables may be distinguished. Firstly, if the aim is to separate variables that are highly, but negatively, correlated each cluster must be defined locally around a latent variable that has the same orientation as the variables in the cluster. On the contrary, if the aim is to group together correlated variables in the same cluster, whatever the sign of the correlation coefficient, each cluster is to be defined directionally around a new axis.

Both cases are associated with a maximization problem [17]. The criterion to be maximized (1) involves the covariance between the variables and the latent variables for local groups:

$$S = \sum_{k=1}^{K} \sum_{j=1}^{p} \delta_{kj} cov\left(x_j, t_k\right) \quad with \quad t_k^t t_k = 1 \qquad (1)$$

For directional groups, the criterion in (2) involves the squared covariance:

$$T = \sum_{k=1}^{K} \sum_{j=1}^{p} \delta_{kj} cov^2\left(x_j, t_k\right) \quad with \quad t_k^t t_k = 1 \qquad (2)$$

In (1) and (2), *K* stands for the number of groups in the partition $\{G_1, \ldots, G_K\}$. x_j represents the jth variable and t_k the latent variable associated with the kth group. δ_{kj} is an indicator equal to 1 if the variable *j* belongs to the group *k*, and equal to 0 otherwise.

The case of directional groups was considered in this case study. It is noteworthy that, in this (2), the optimum value of *T* is obtained when the latent variable t_k within each cluster G_k is the first normalized principal component of the variables belonging to this cluster.

The maximization of the CLV criteria for *K* fixed is achieved by an iterative alternating procedure whose monotonicity can be proven. Moreover, partitions of growing size, from one (all the variables into only one group) to *p* groups (each variable in its own group) may be extracted in the course of an hierarchical algorithm. The CLV approach has been implemented in an R-package, ClustVarLV, freely available on the CRAN website [18].
