# Supplementary material for: Estimating biological accuracy of DSM for attention deficit/hyperactivity disorder based on multivariate analysis for small samples
Source: PeerJ. 2019 Jun 12;7:e7074. doi: 10.7717/peerj.7074 (PMC6571005; doi:10.7717/peerj.7074)
Supplement: Supplemental Information 3 — For emulate independent samples –see main text for details. [file peerj-07-7074-s003.ppt]

## Slide 1
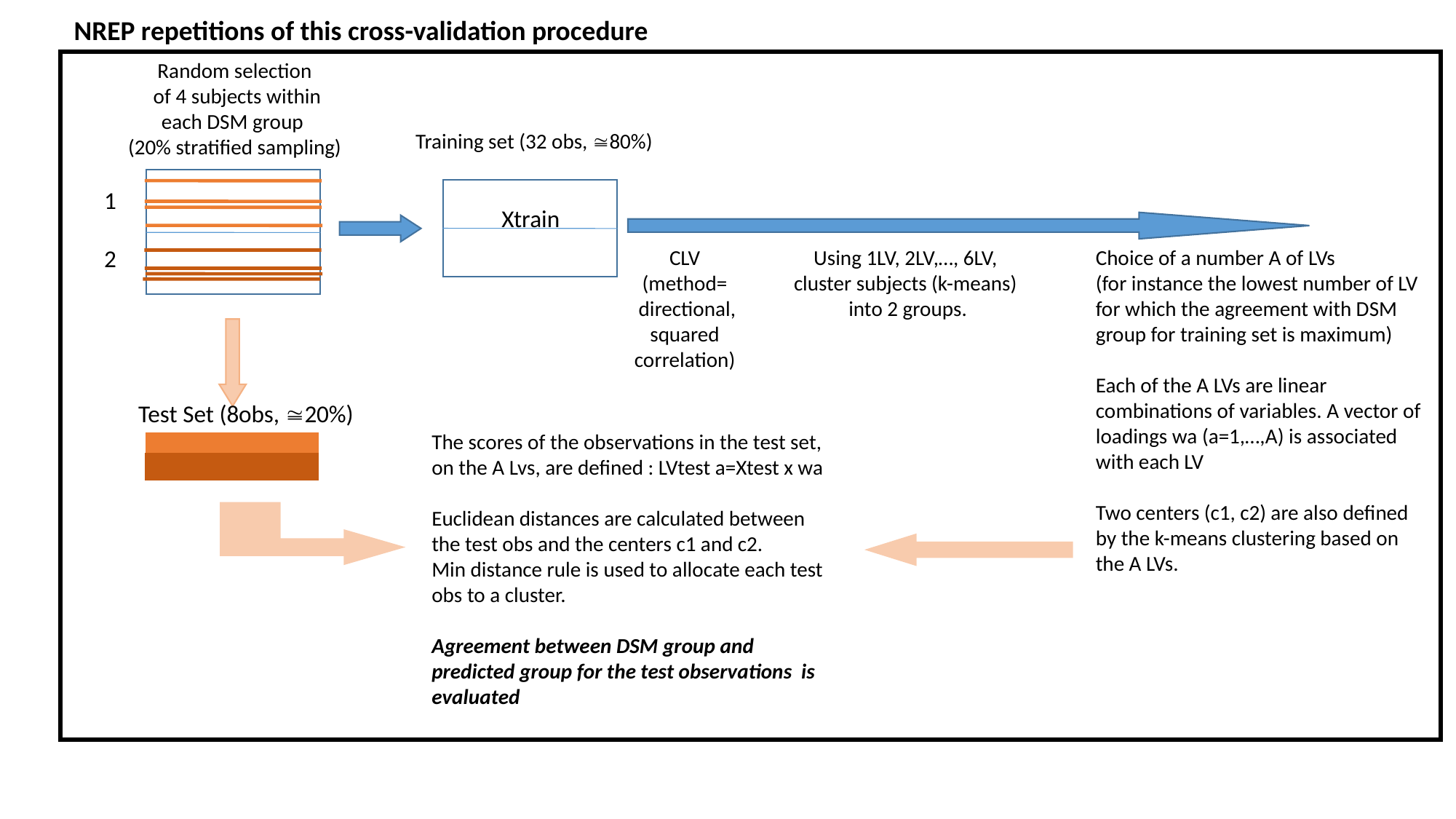

NREP repetitions of this cross-validation procedure
Random selection
 of 4 subjects within
 each DSM group
(20% stratified sampling)
Training set (32 obs, 80%)
1
2
Xtrain
CLV
(method=
 directional,
 squared
correlation)
Using 1LV, 2LV,…, 6LV,
cluster subjects (k-means)
into 2 groups.
Choice of a number A of LVs
(for instance the lowest number of LV for which the agreement with DSM group for training set is maximum)
Each of the A LVs are linear combinations of variables. A vector of loadings wa (a=1,…,A) is associated with each LV
Two centers (c1, c2) are also defined by the k-means clustering based on the A LVs.
Test Set (8obs, 20%)
The scores of the observations in the test set, on the A Lvs, are defined : LVtest a=Xtest x wa
Euclidean distances are calculated between the test obs and the centers c1 and c2.
Min distance rule is used to allocate each test obs to a cluster.
Agreement between DSM group and predicted group for the test observations is evaluated
