# Supplementary figures and images for: Estimating biological accuracy of DSM for attention deficit/hyperactivity disorder based on multivariate analysis for small samples
Source: PeerJ. 2019 Jun 12;7:e7074. doi: 10.7717/peerj.7074 (PMC6571005; doi:10.7717/peerj.7074)

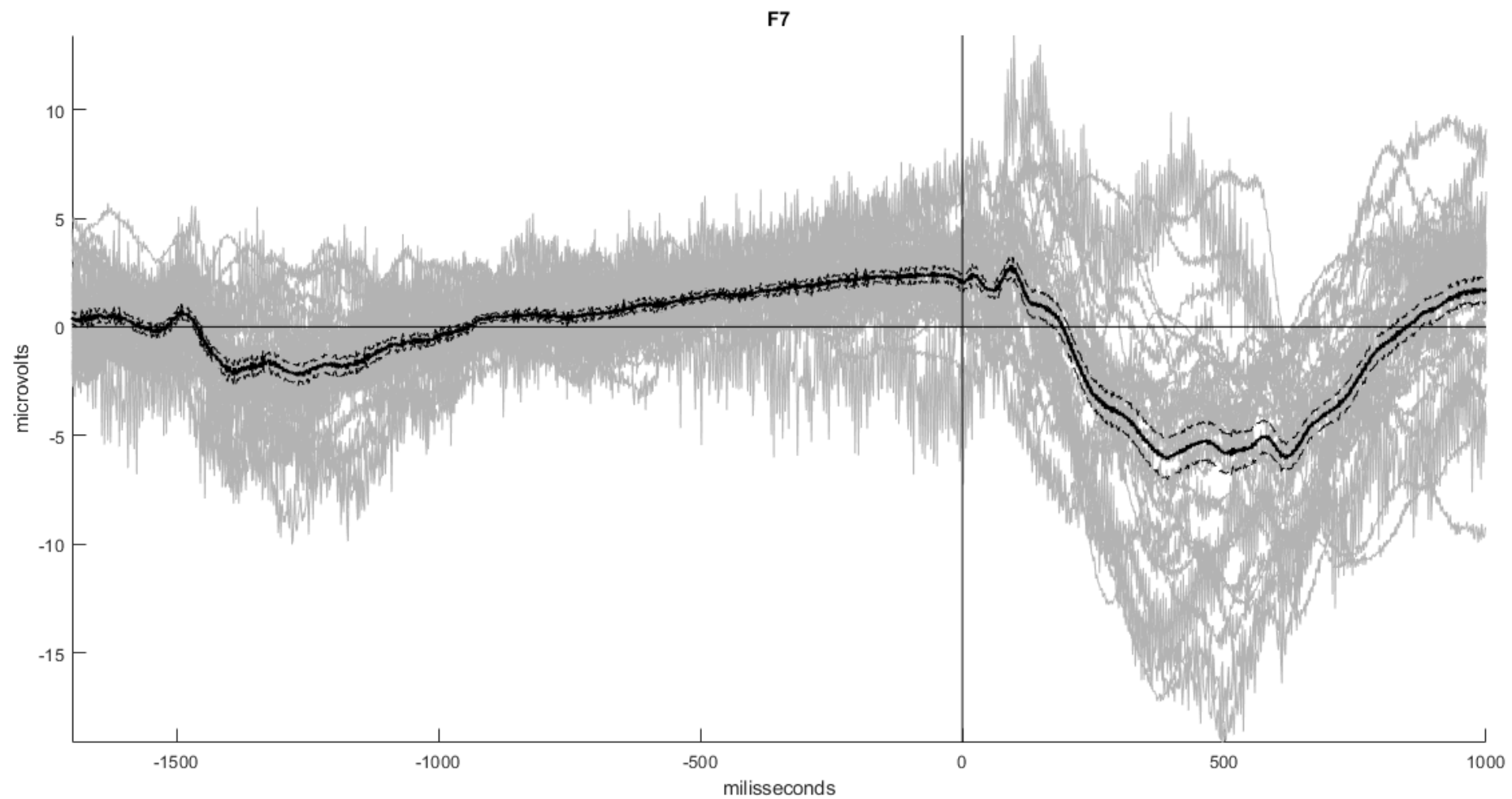

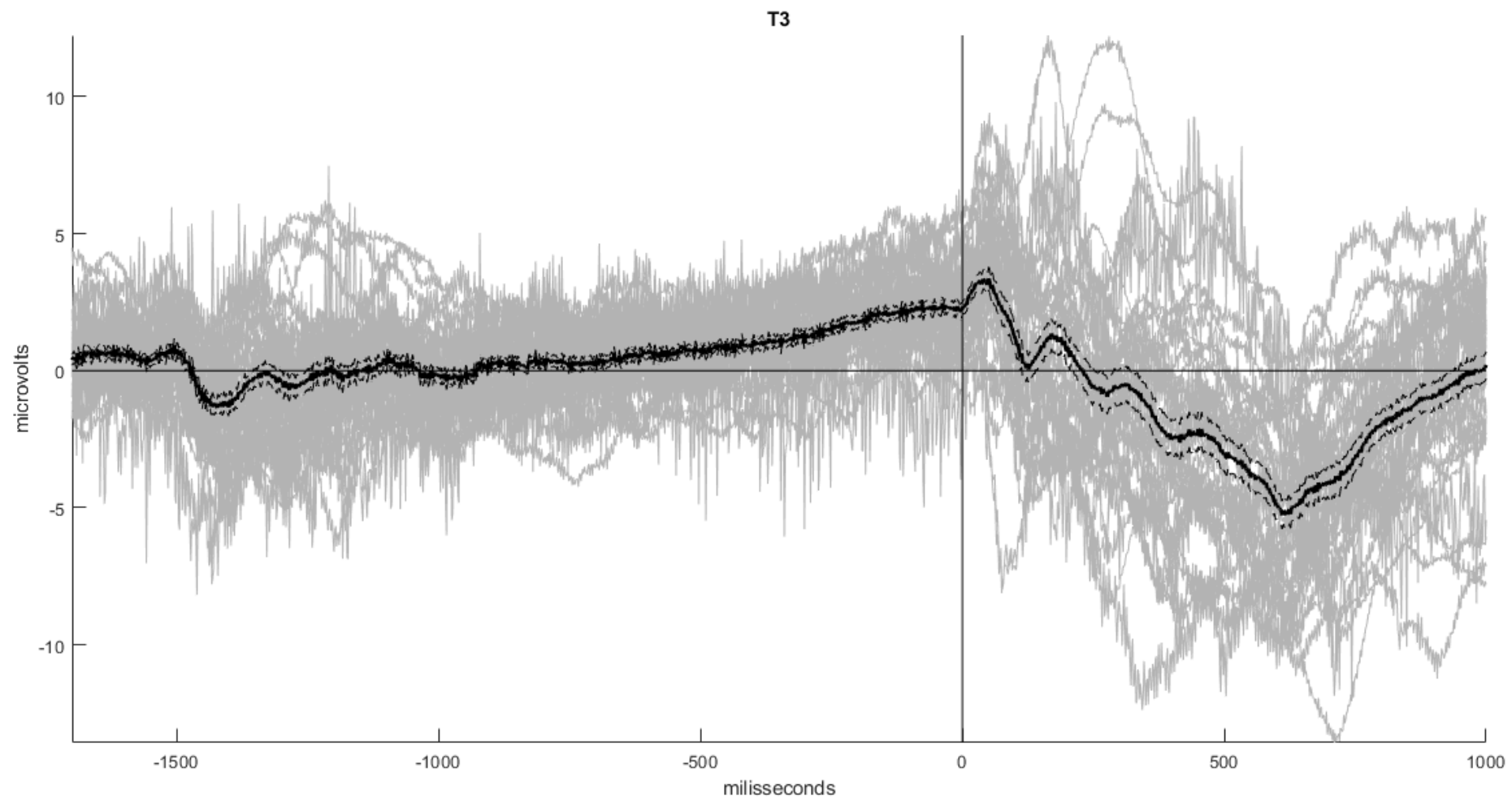

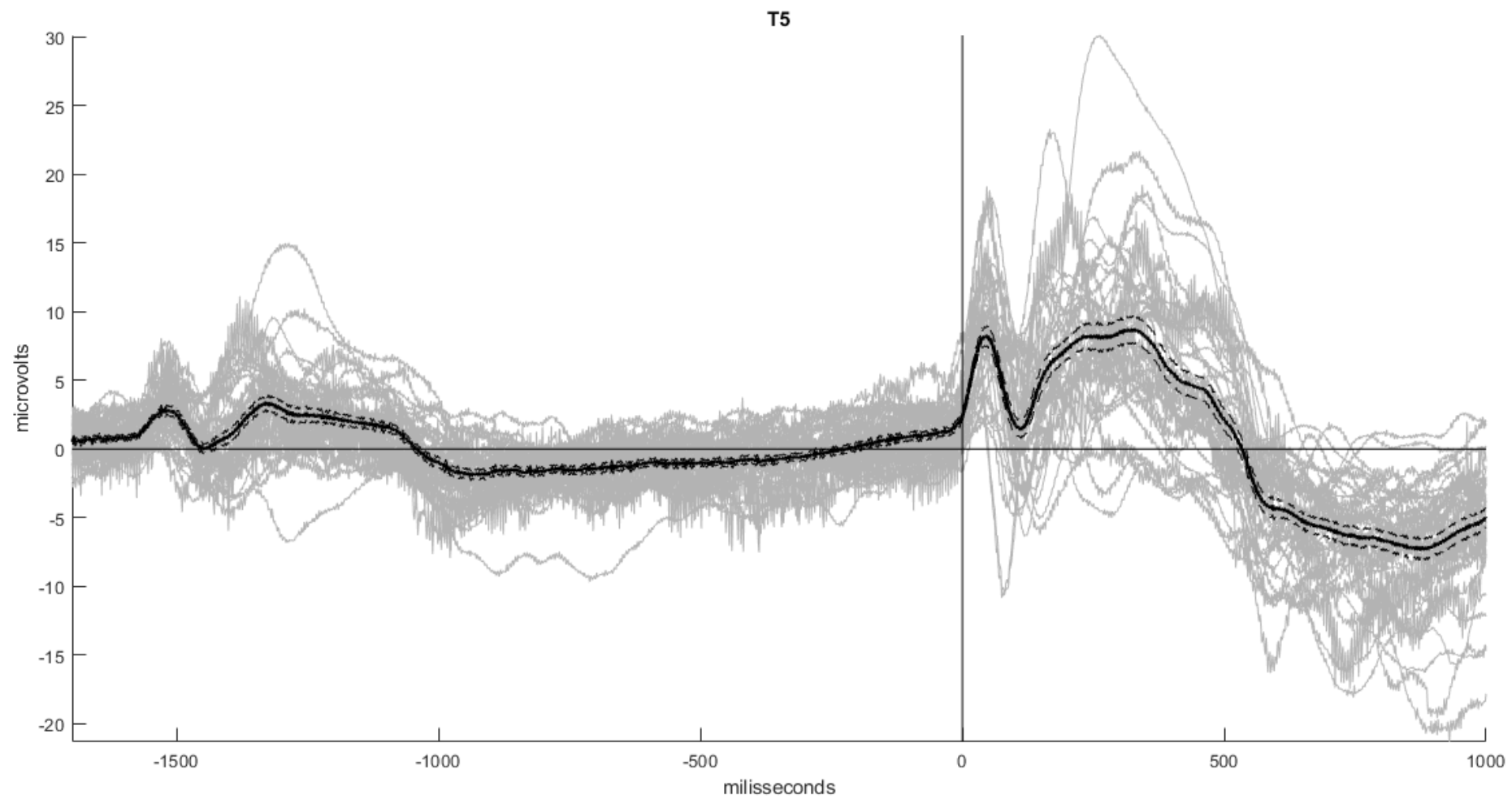

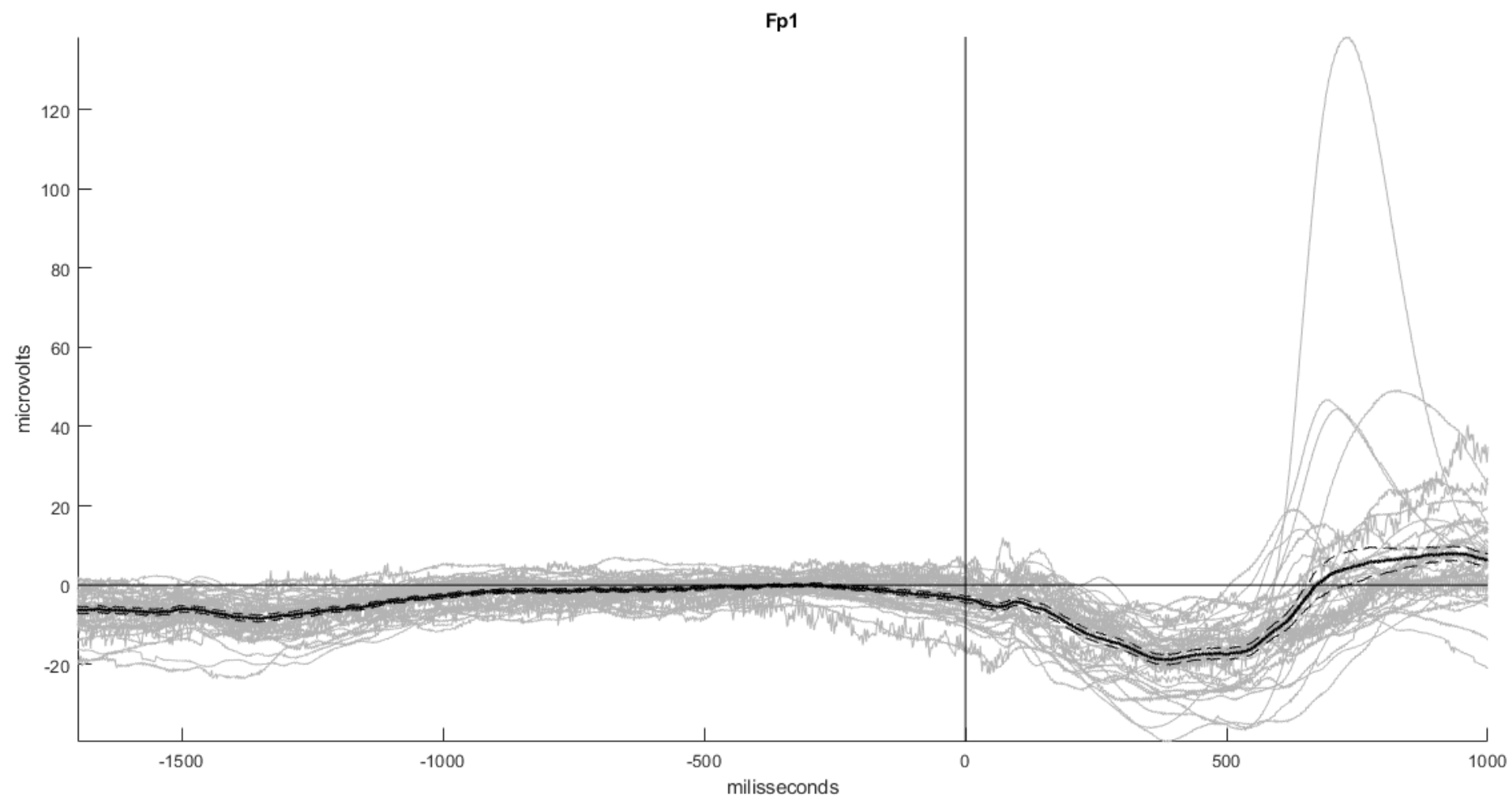

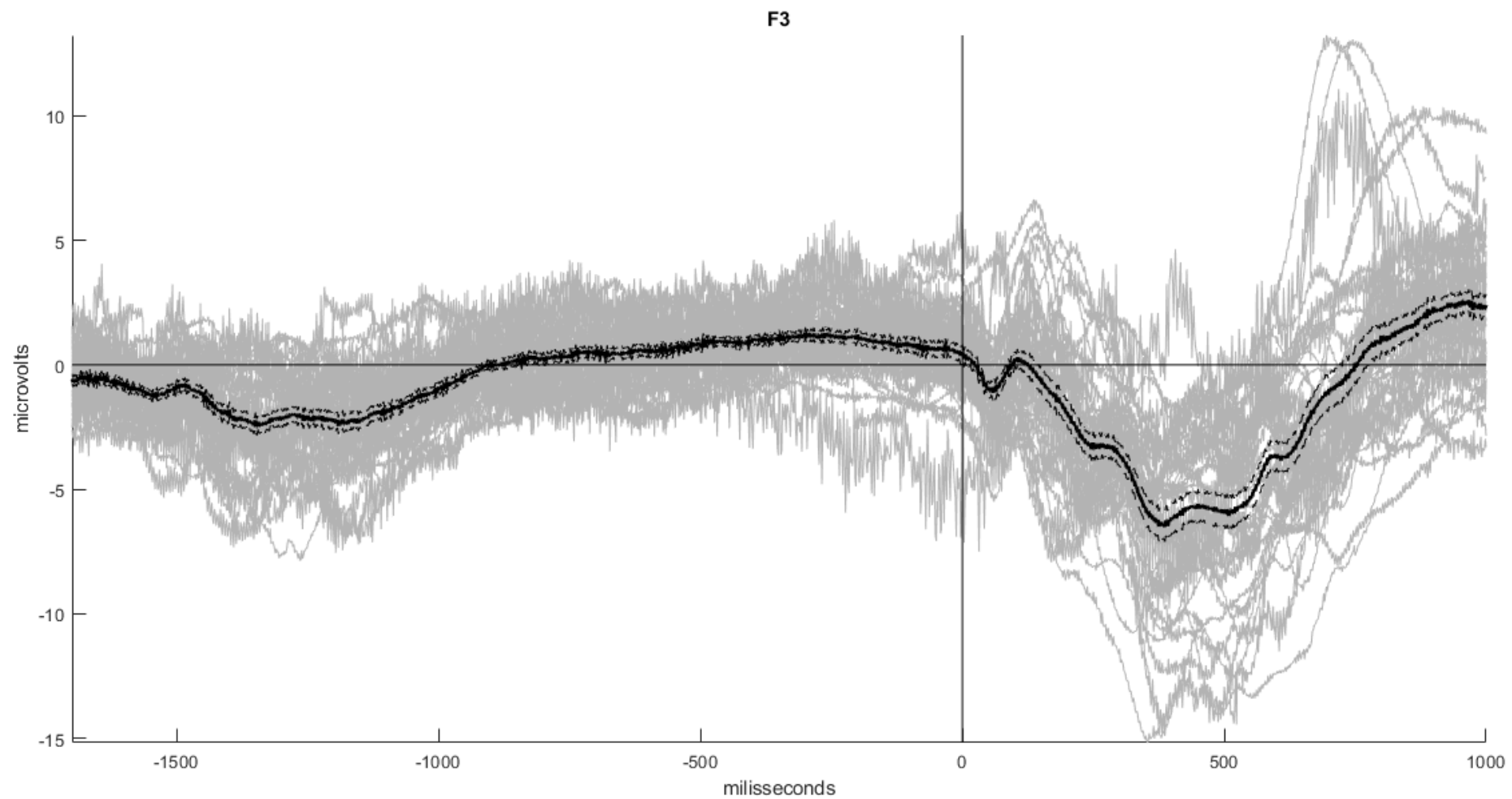

**C3-C4**

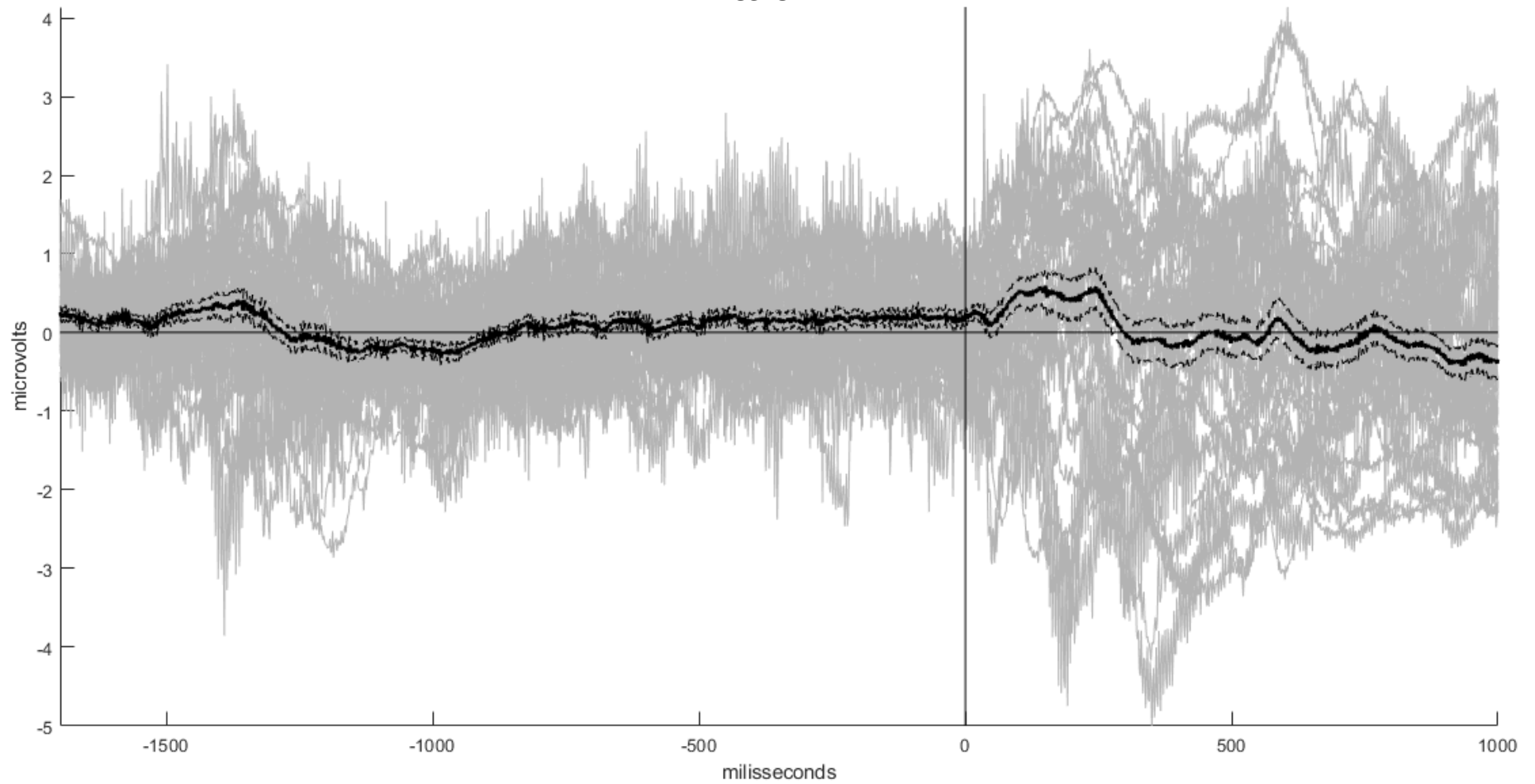

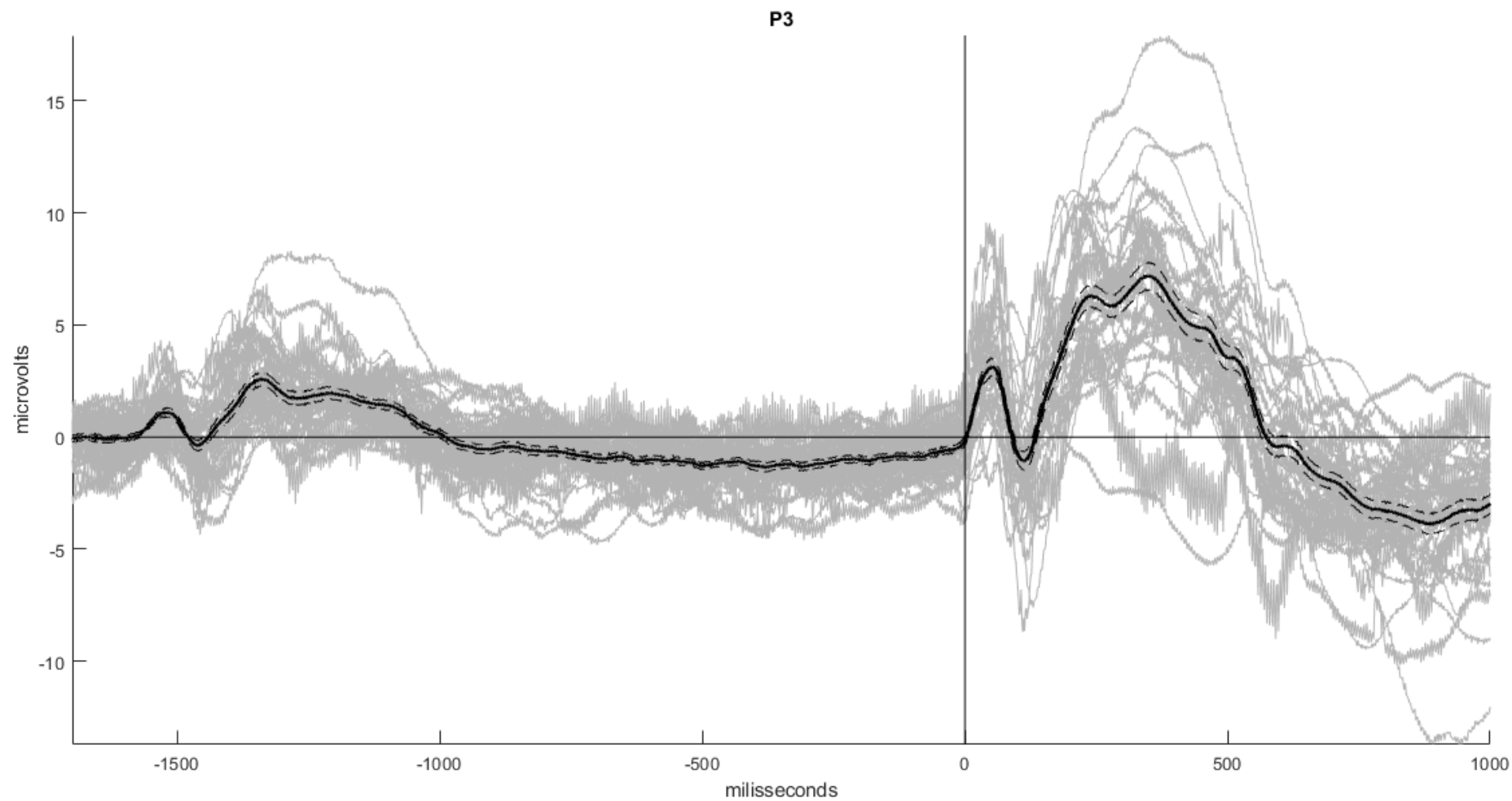

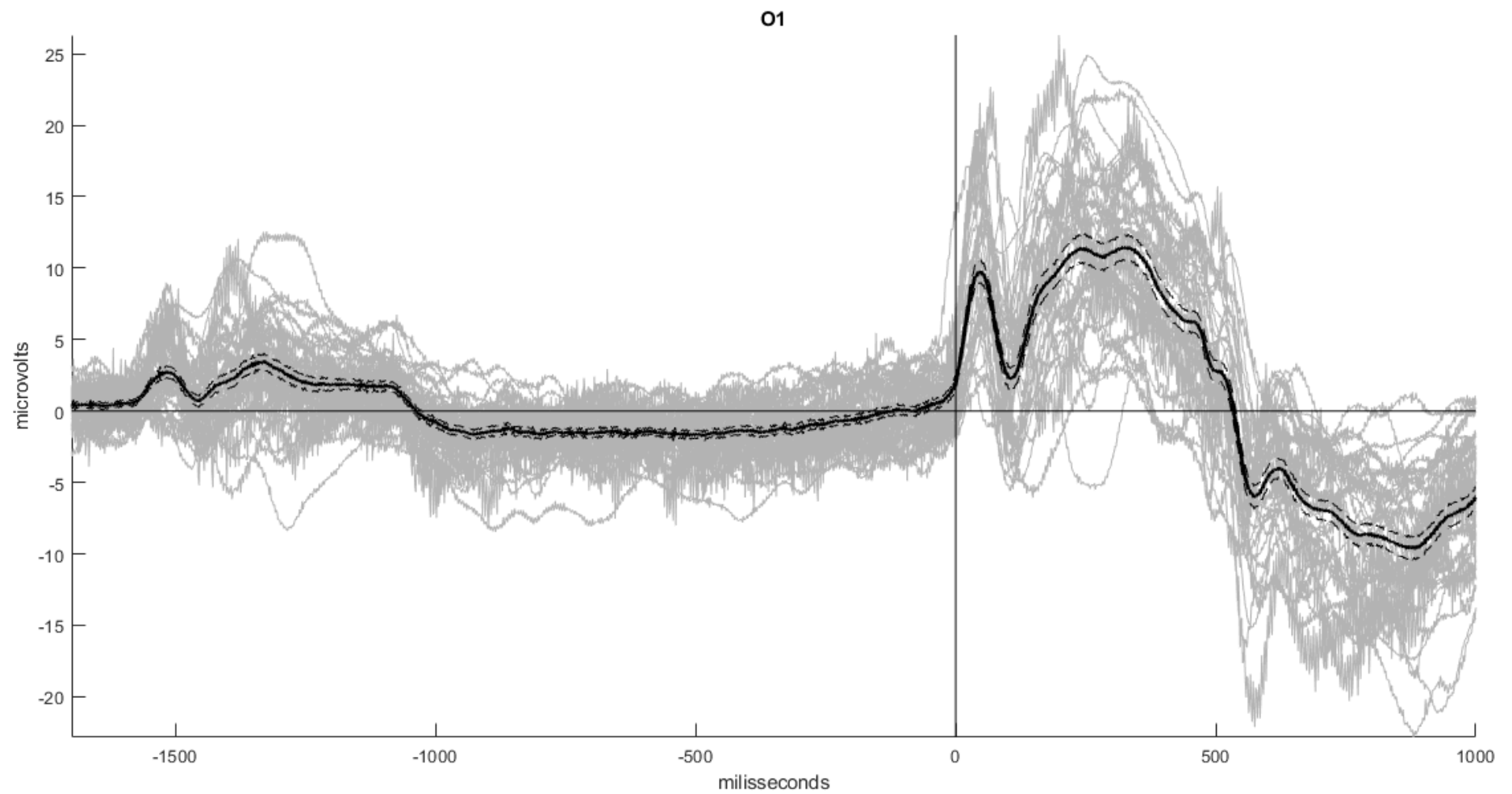

F8

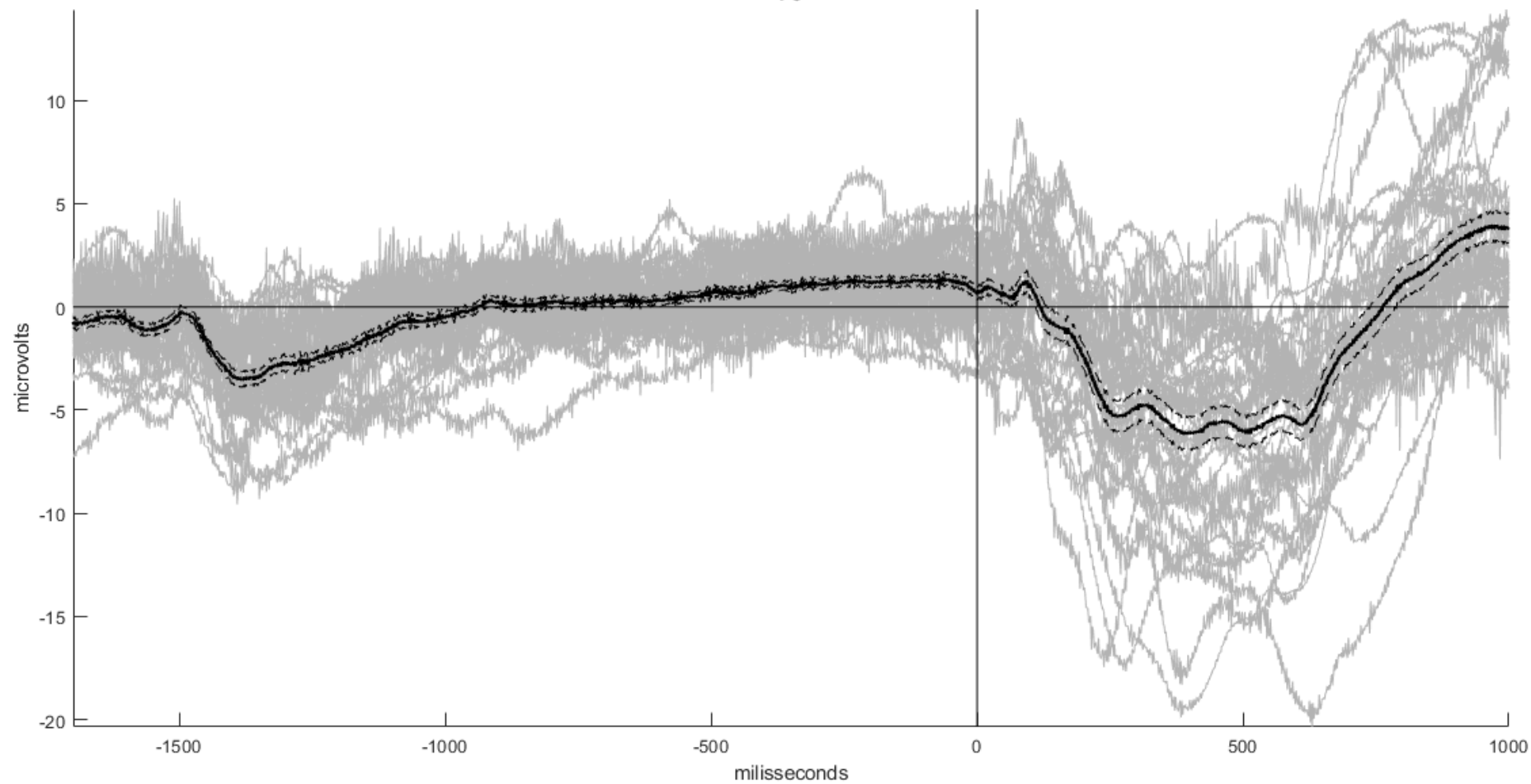

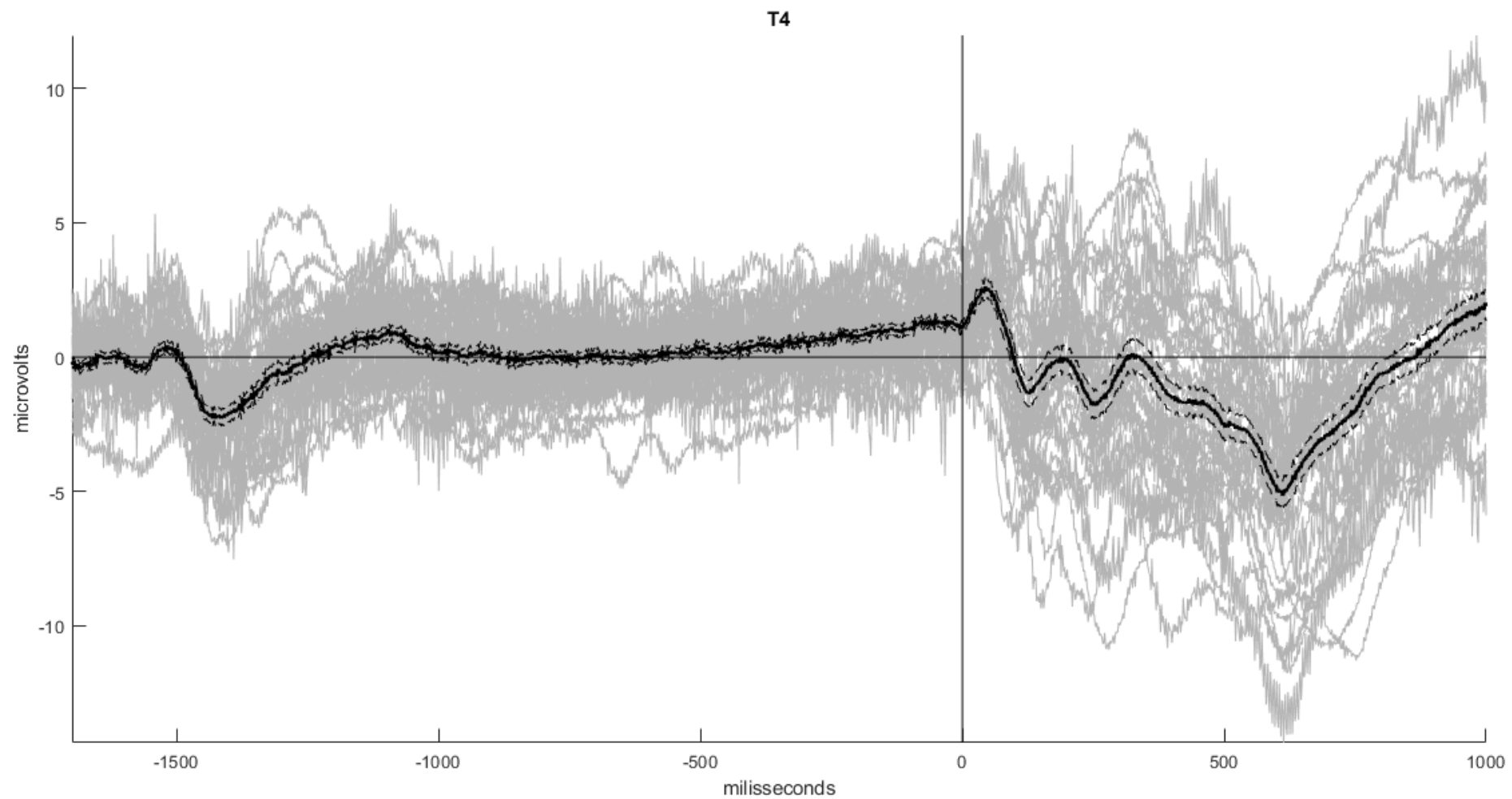

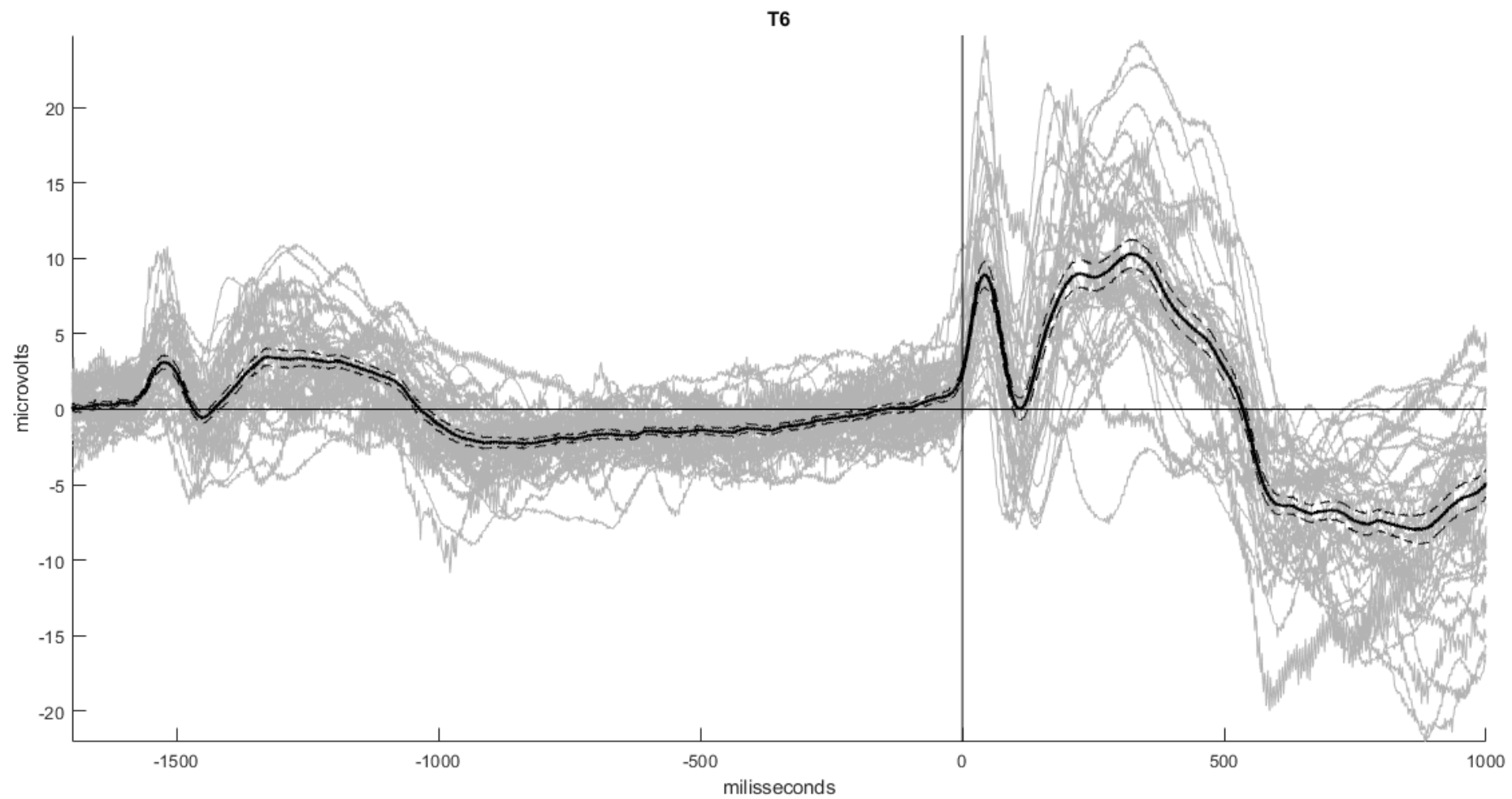

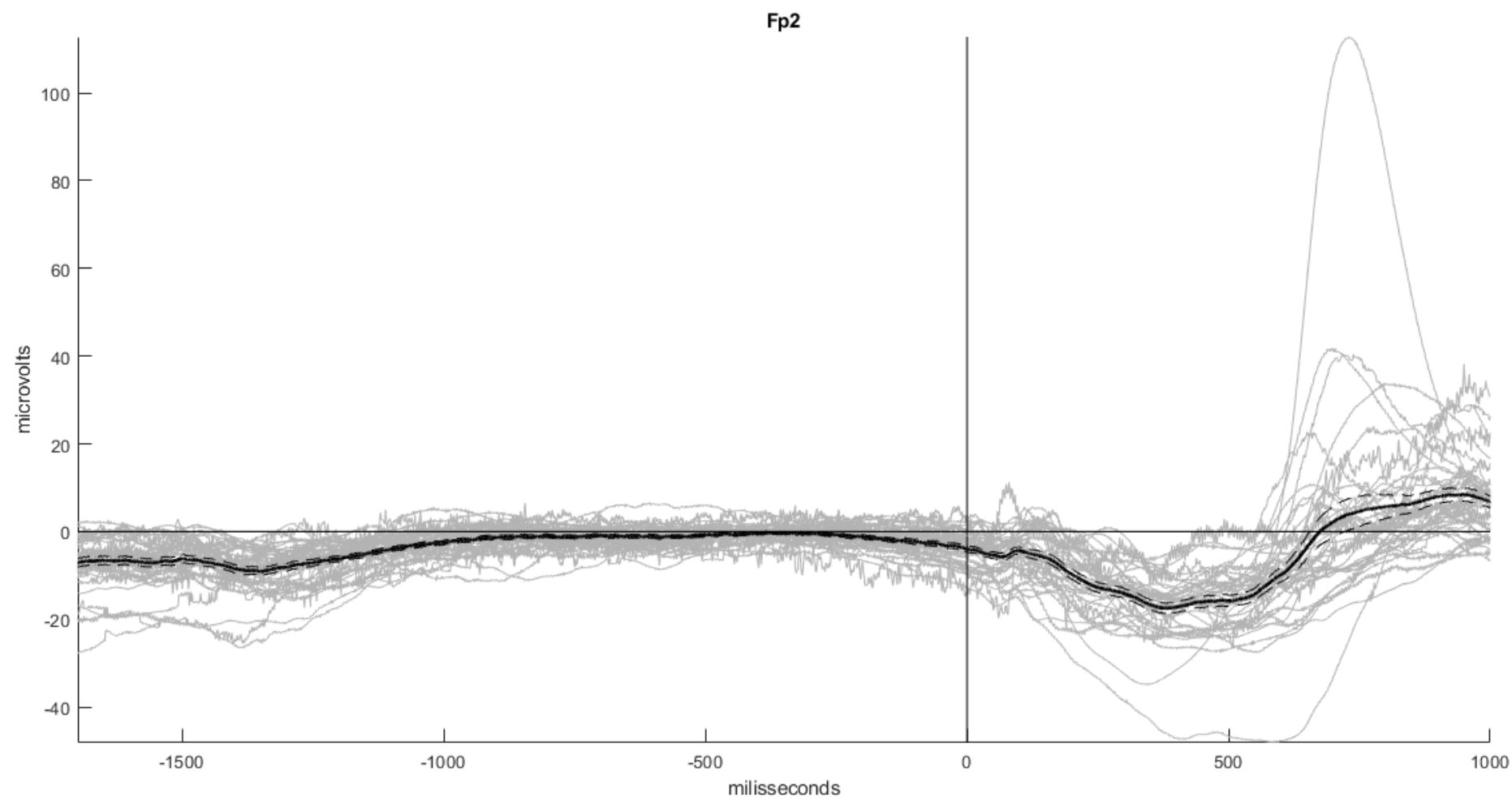

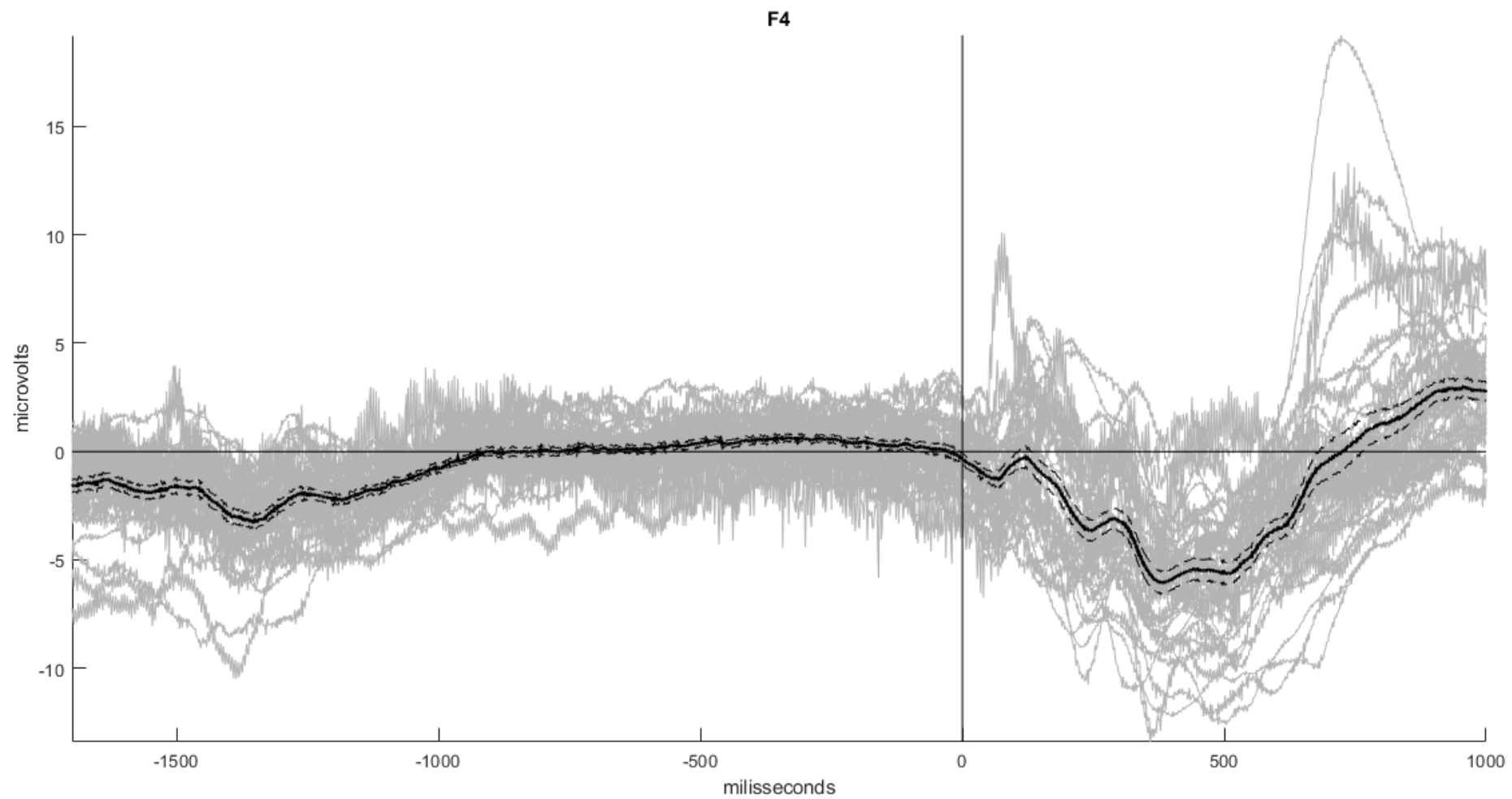

P4

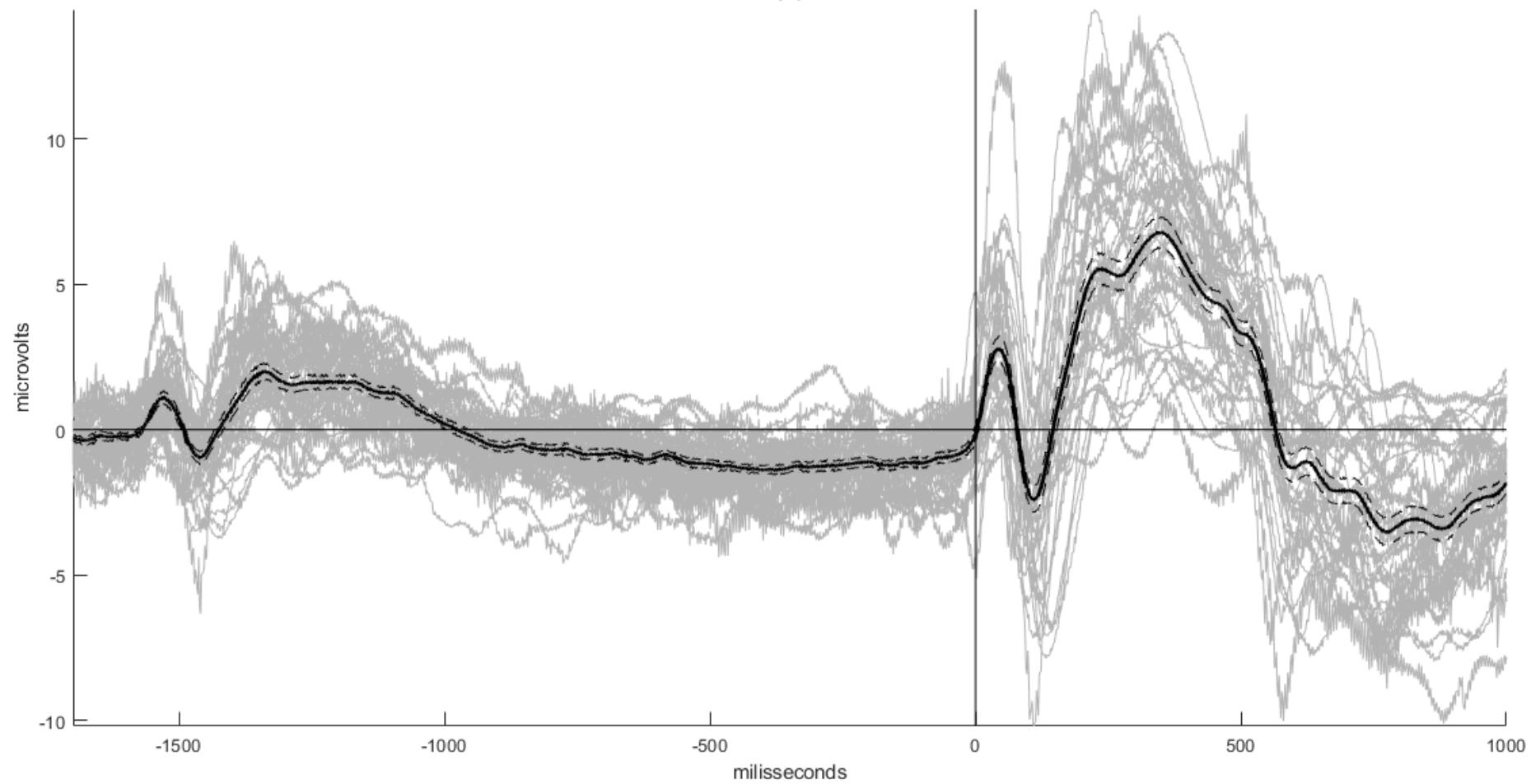

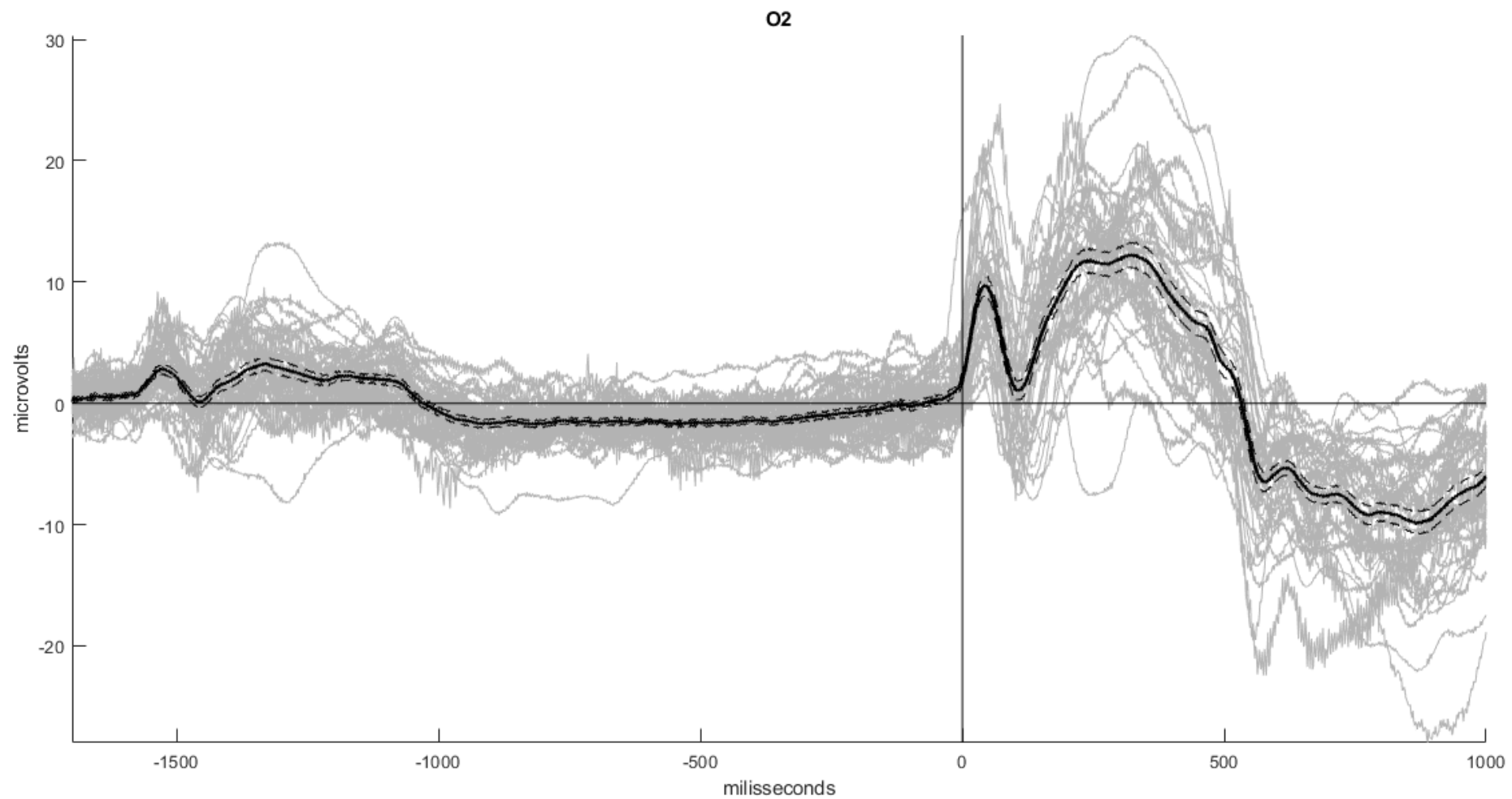

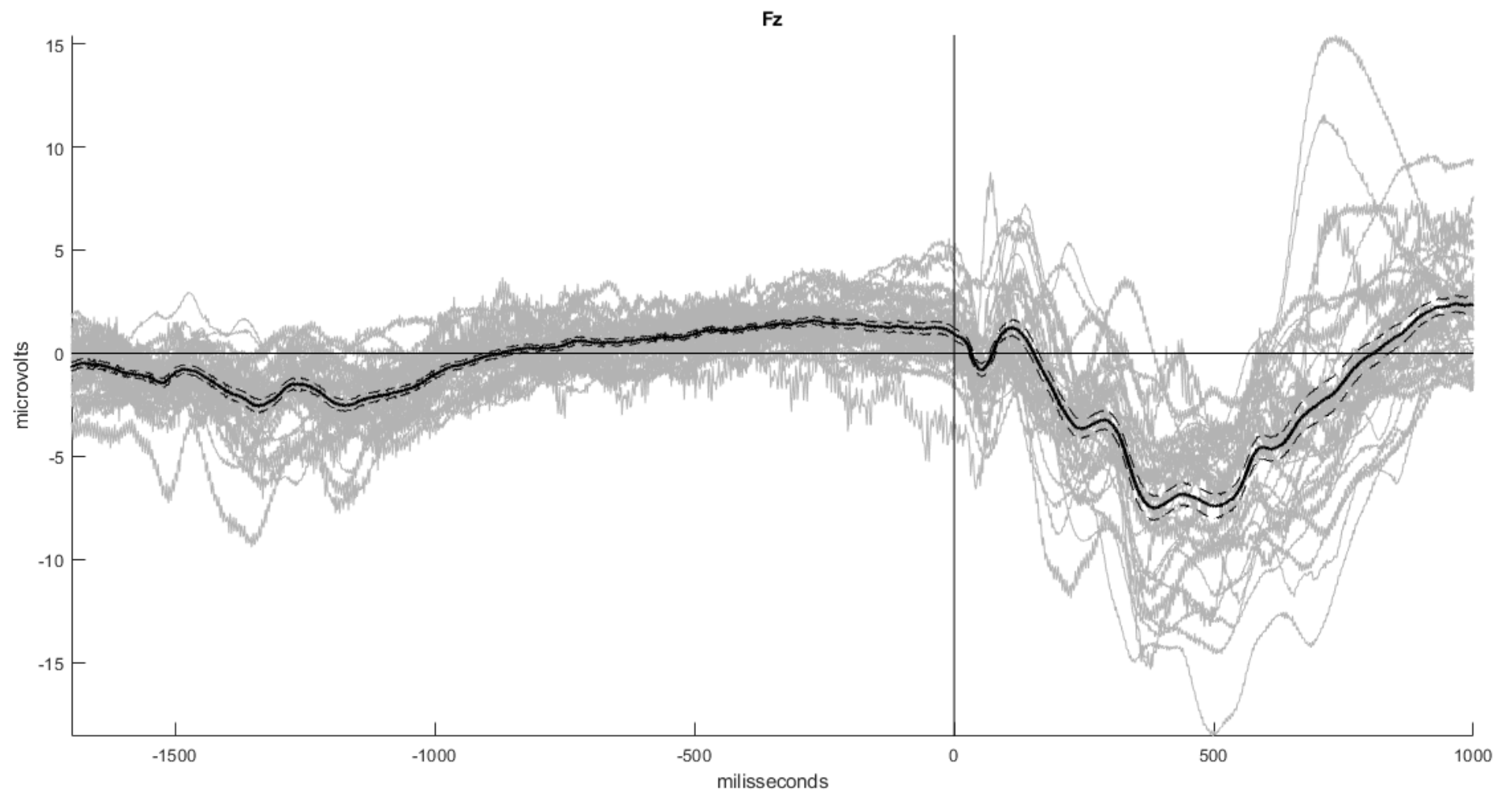

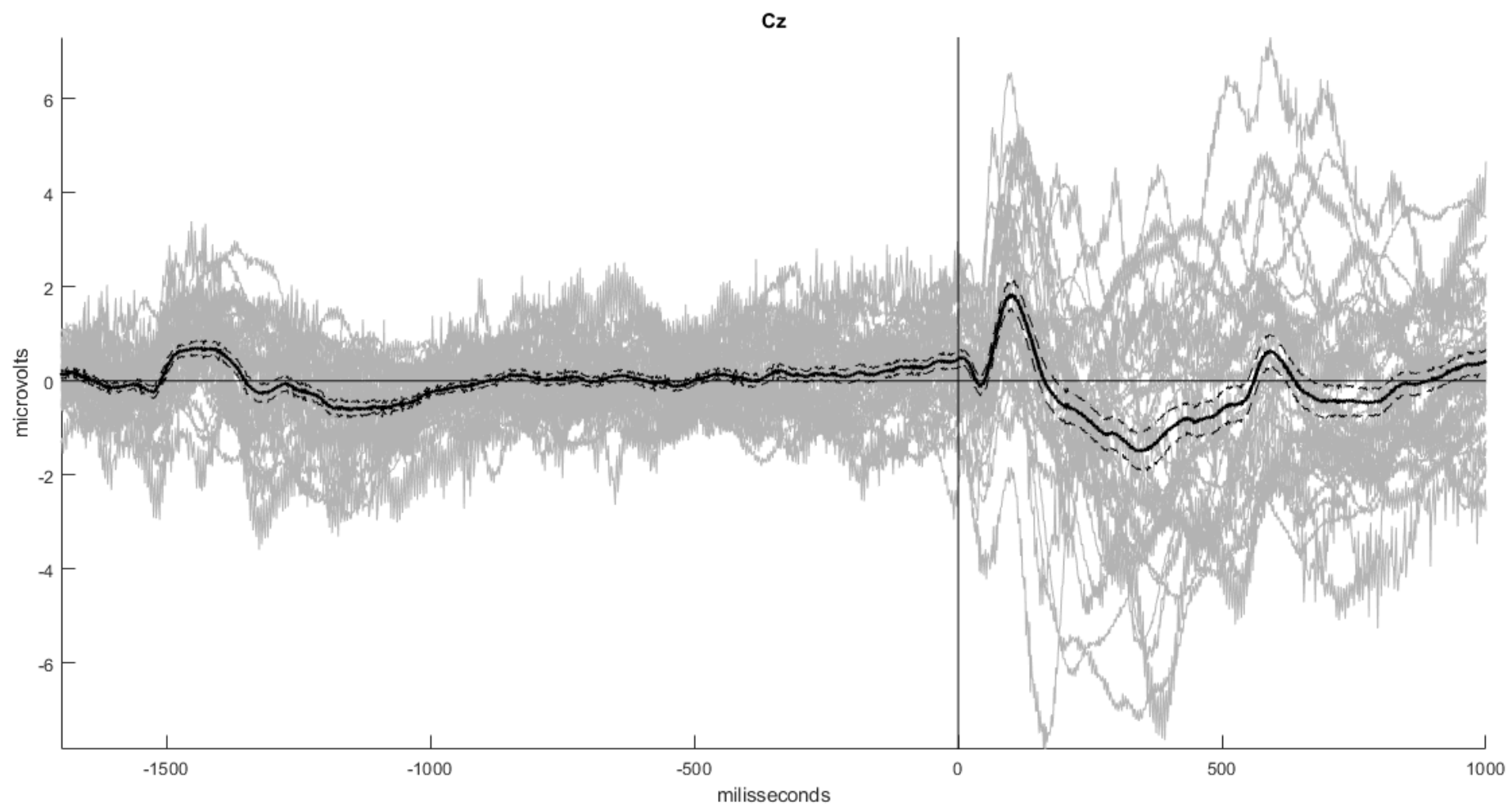

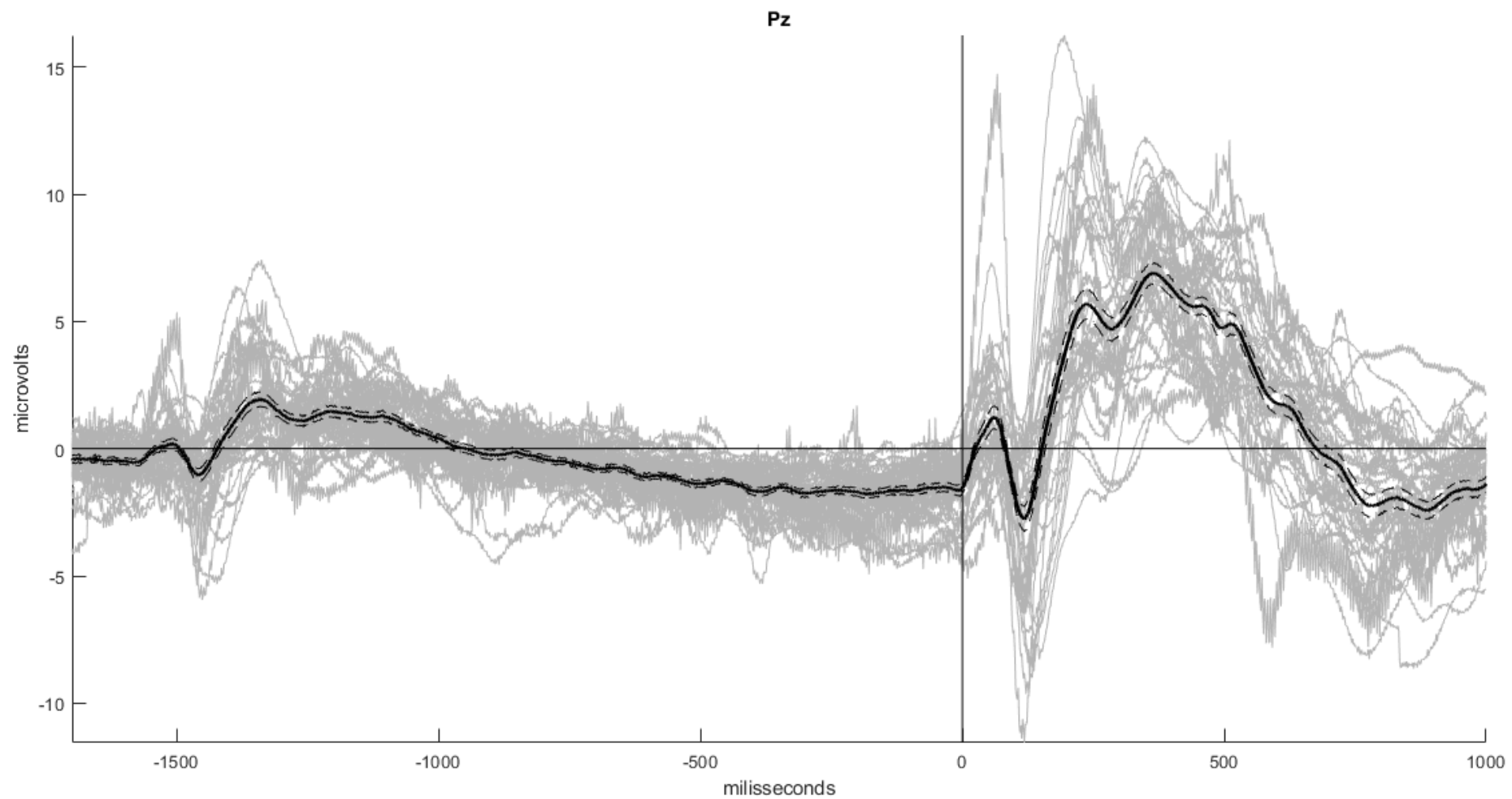

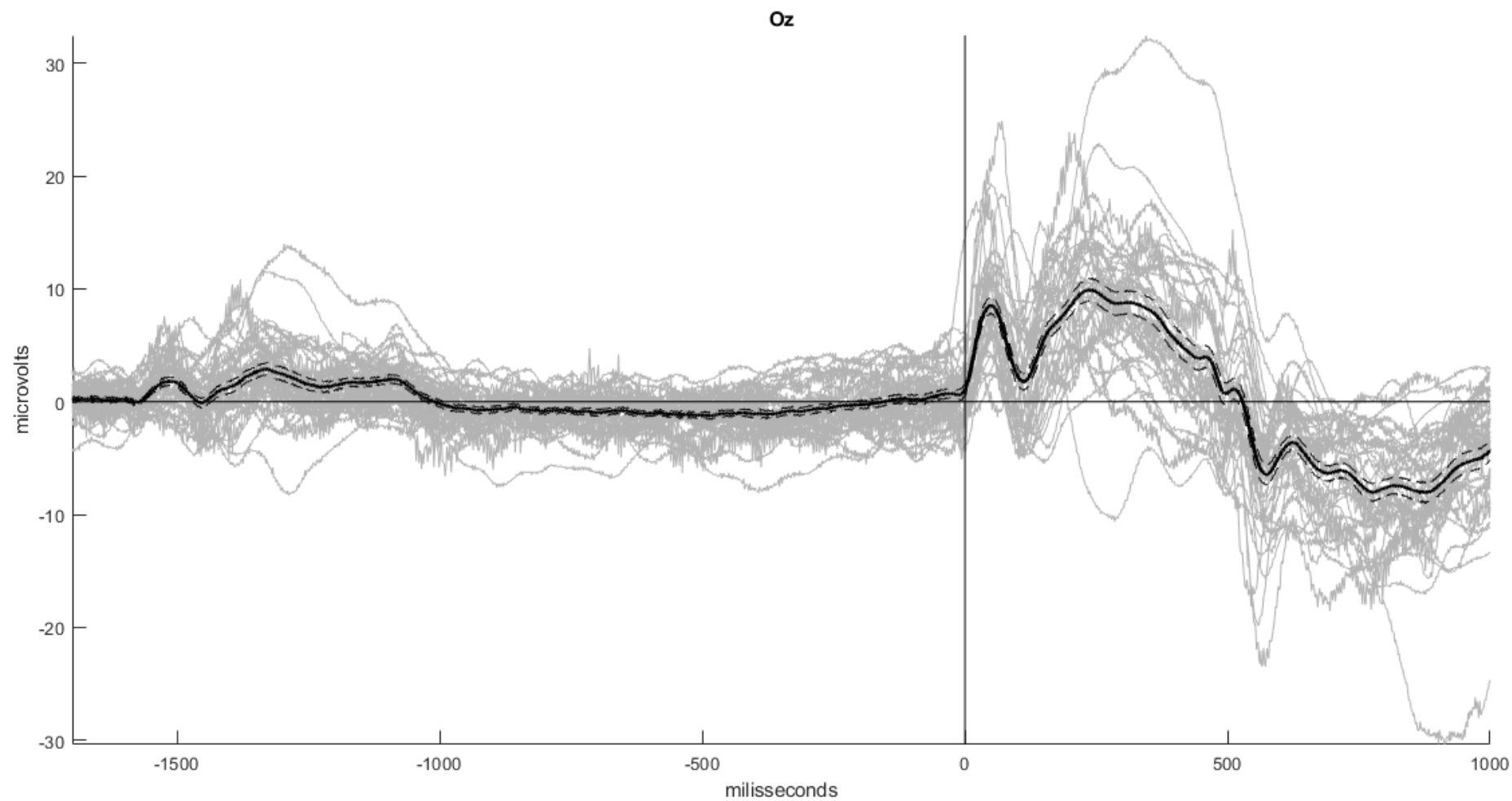

Supplement: Figure S1 — Individual waves from the sample for each channel (all conditions), by page. The waves (gray) are superimposed to their mean values (black thick line) and their standard error of mean (black thin dotted line). Amplitude in microvolts. Waves related to all ANT conditions indistinctly. [file peerj-07-7074-s006.pdf]

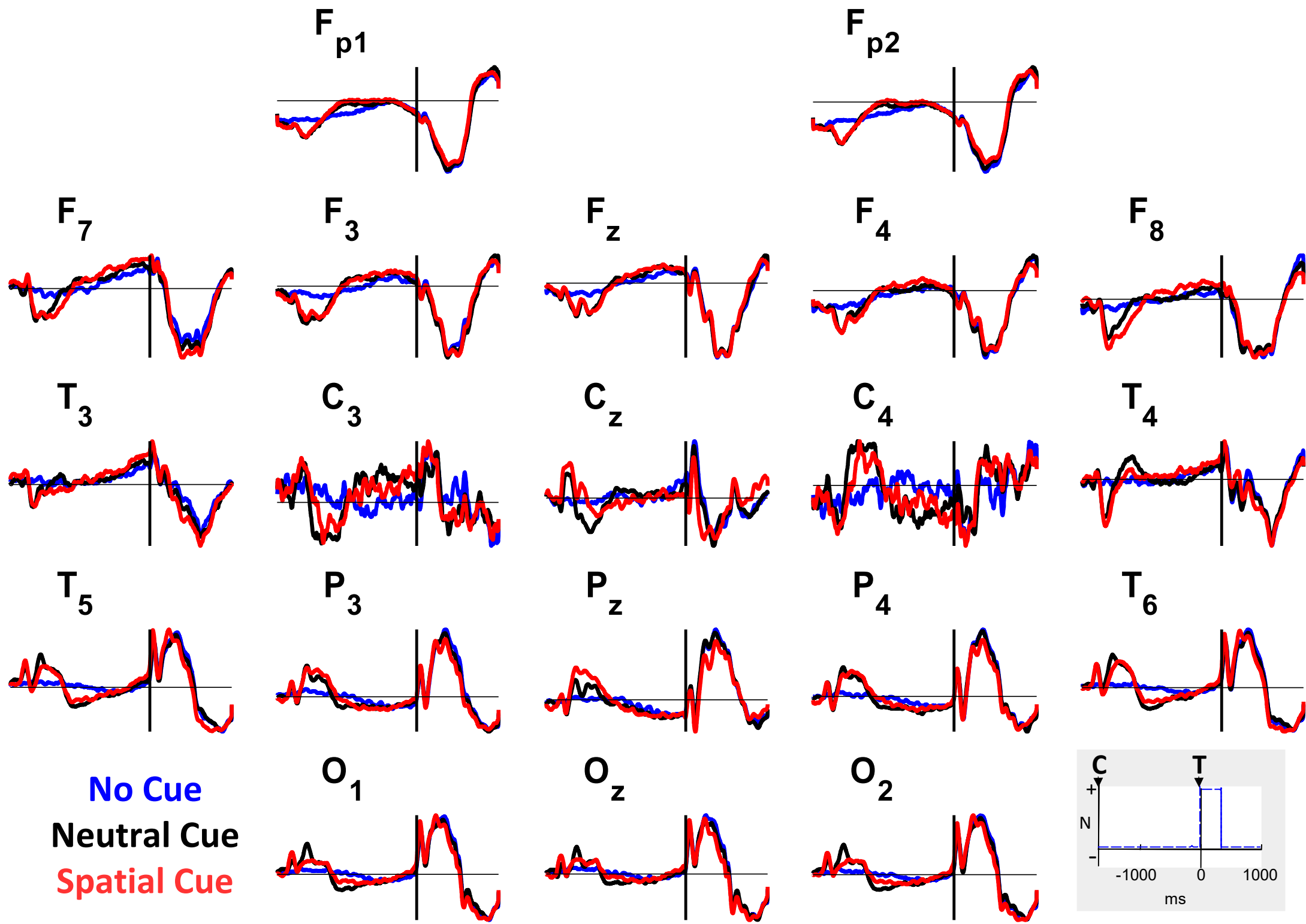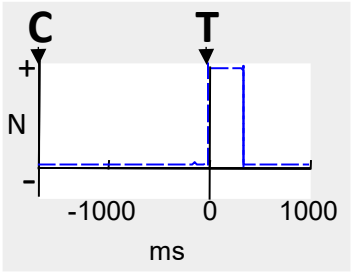

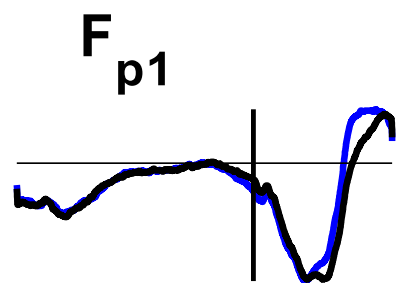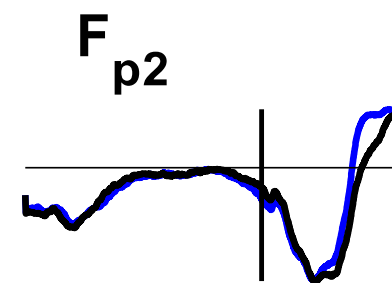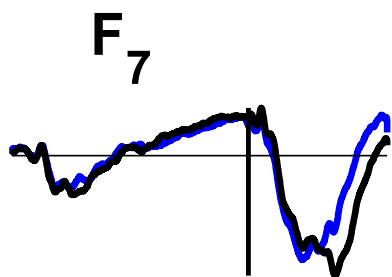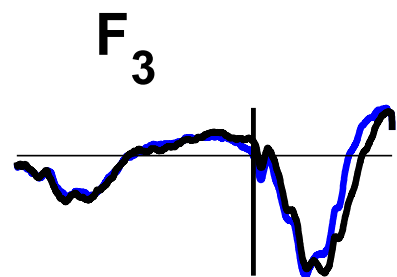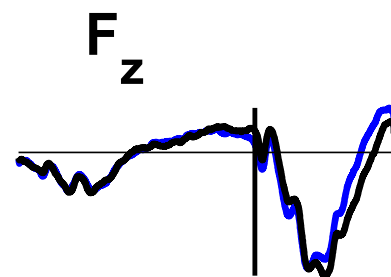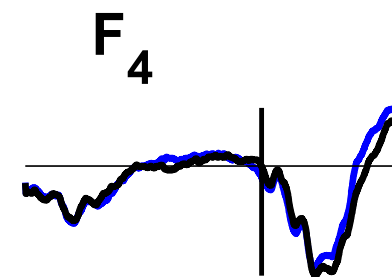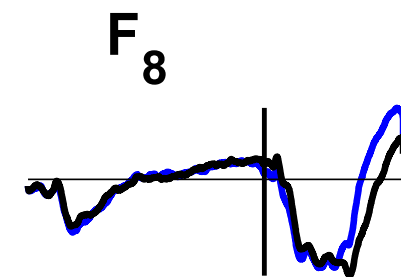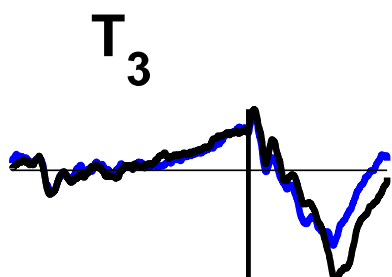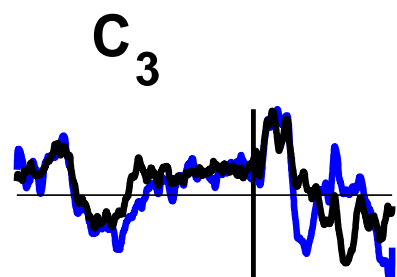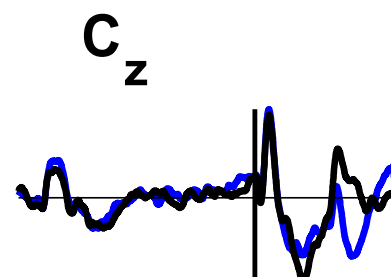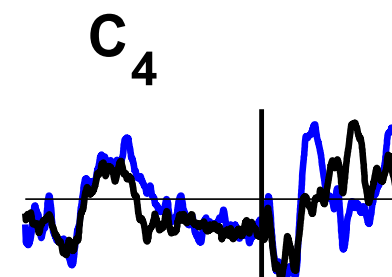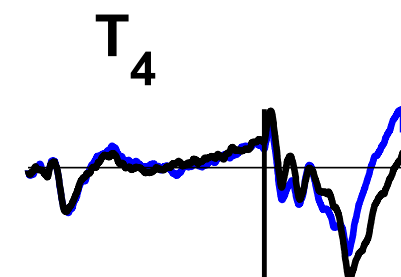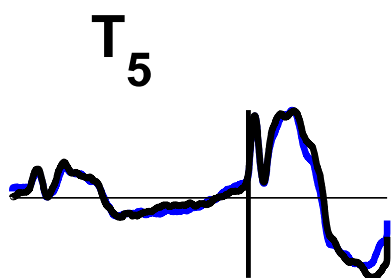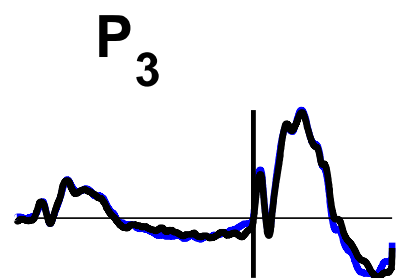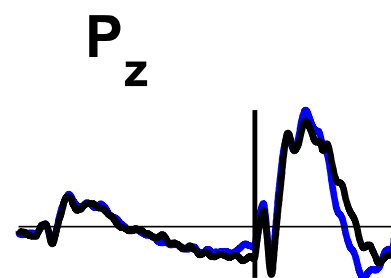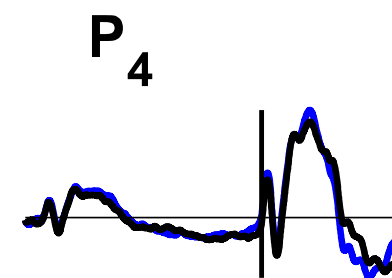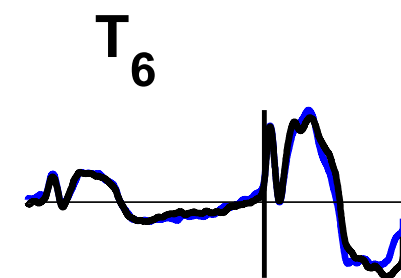

**Congruent**  
**Target**  
**Incongruent**  
**Target**

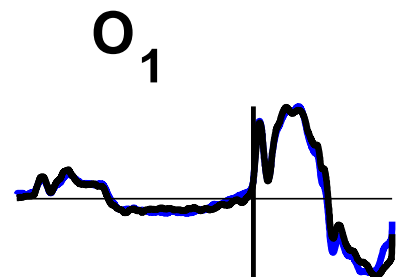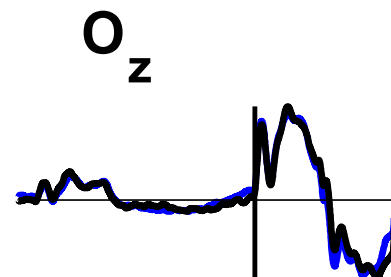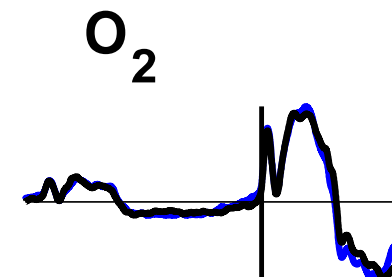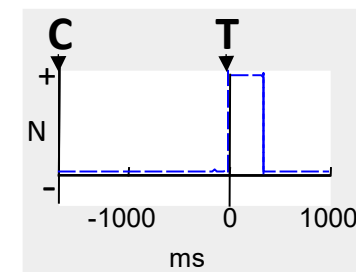

Supplement: Figure S2 — Sample means (n = 39) of No Cue (blue), Neutral Cue (black) and Spatial Cue (red) -related potentials are superimposed in (A), and Congruent (blue) and incongruent (black) -related target conditions are superimposed in (B). See caption of figure 1 for more details. [file peerj-07-7074-s007.pdf]
